# Supplementary figures and images for: Prickle and Ror modulate Dishevelled-Vangl interaction to regulate non-canonical Wnt signaling during convergent extension in Xenopus
Source: eLife. 2026 Apr 30;12:RP91199. doi: 10.7554/eLife.91199 (PMC13132548; doi:10.7554/eLife.91199)

|                |   |   |   |   |   |   |
|----------------|---|---|---|---|---|---|
| EGFP-Vangl2    | - | + | - | - | + | - |
| EGFP-Vangl2-RH | - | - | + | - | - | + |
| flag-Pk        | + | + | + | + | + | + |

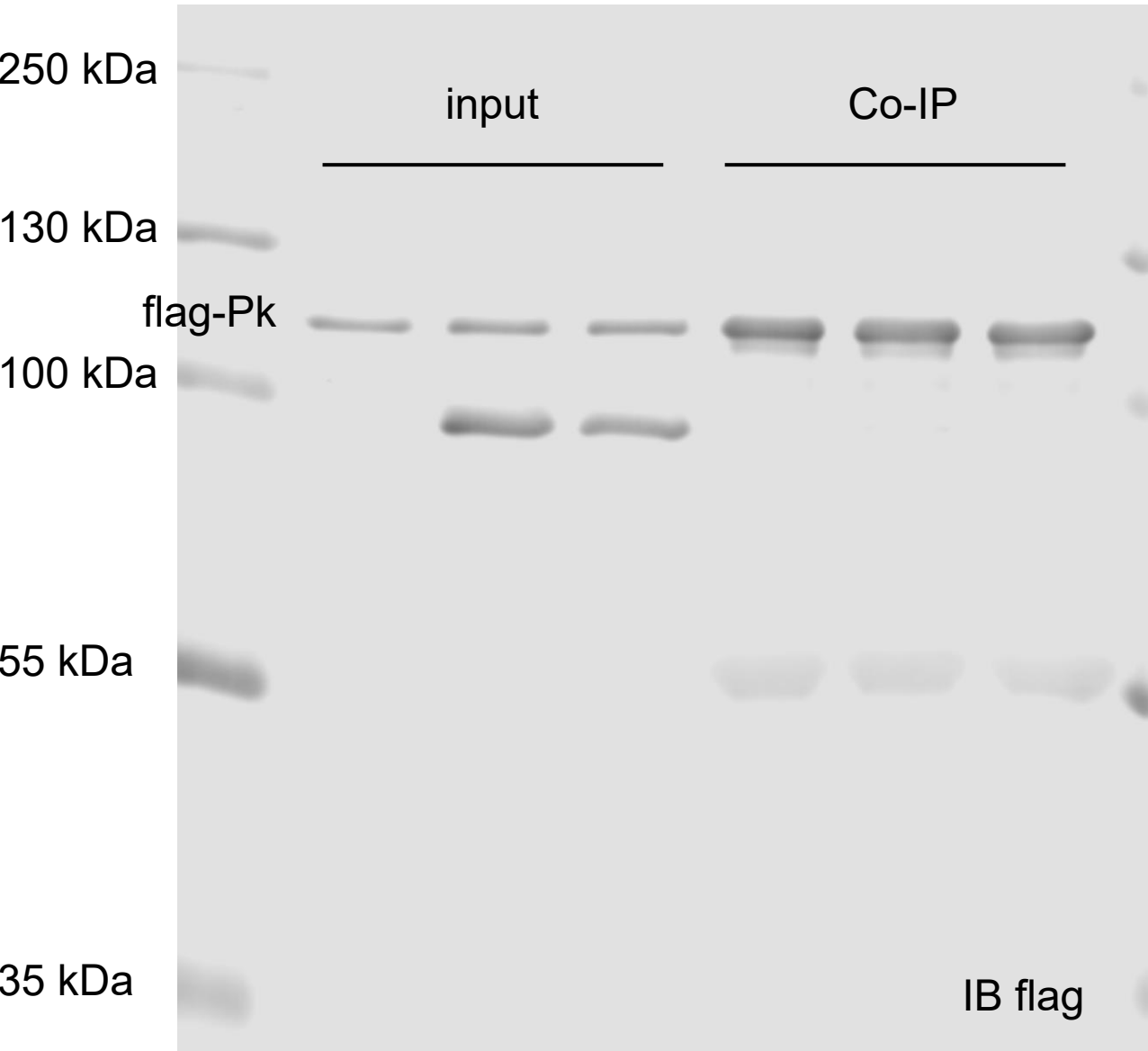

|                |   |   |   |   |   |   |
|----------------|---|---|---|---|---|---|
| EGFP-Vangl2    | - | + | - | - | + | - |
| EGFP-Vangl2-RH | - | - | + | - | - | + |
| flag-Pk        | + | + | + | + | + | + |

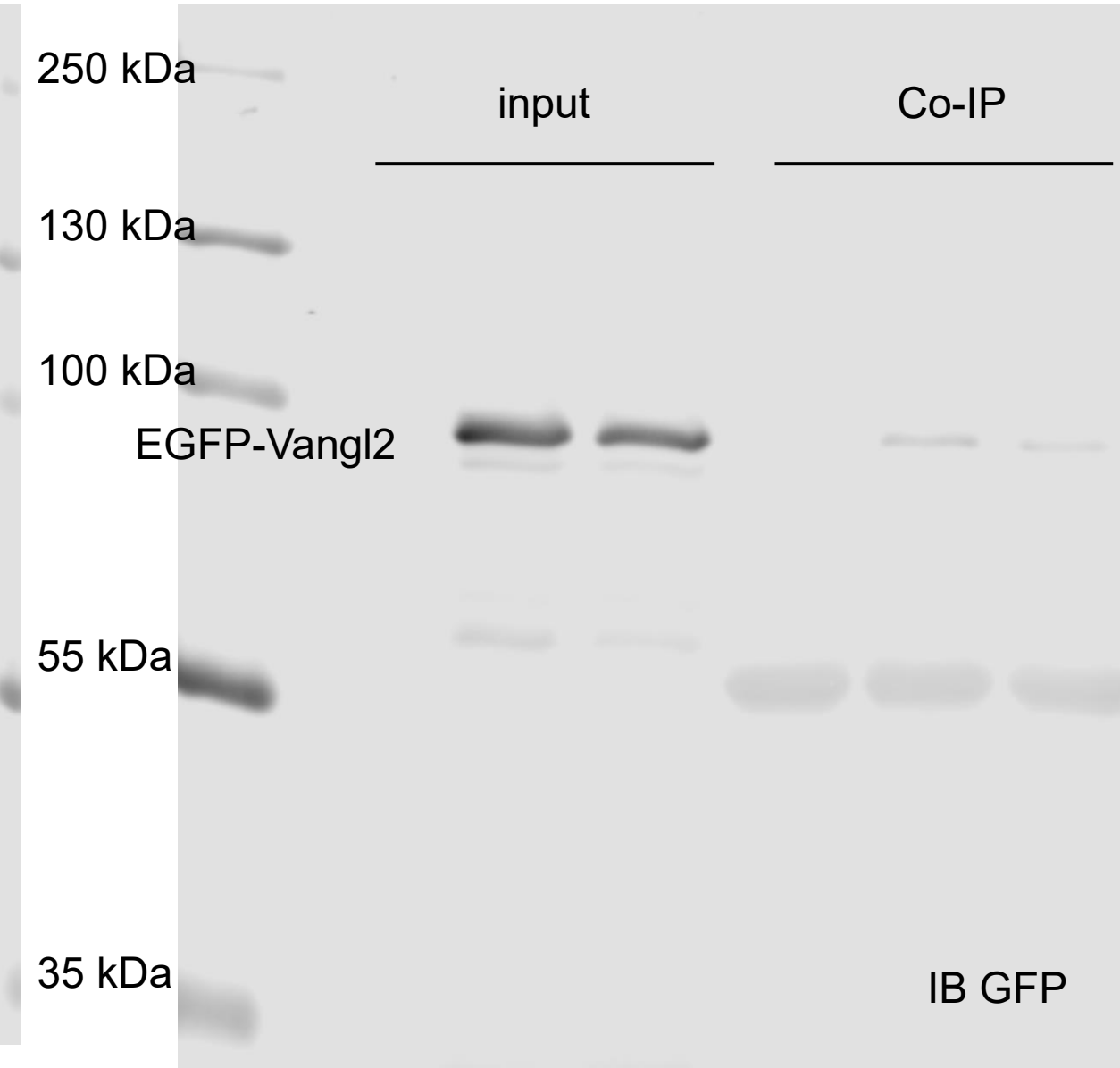

Supplement: Figure 2—source data 1. [file elife-91199-fig2-data1.zip › Figure 2g-source data 1.pdf]

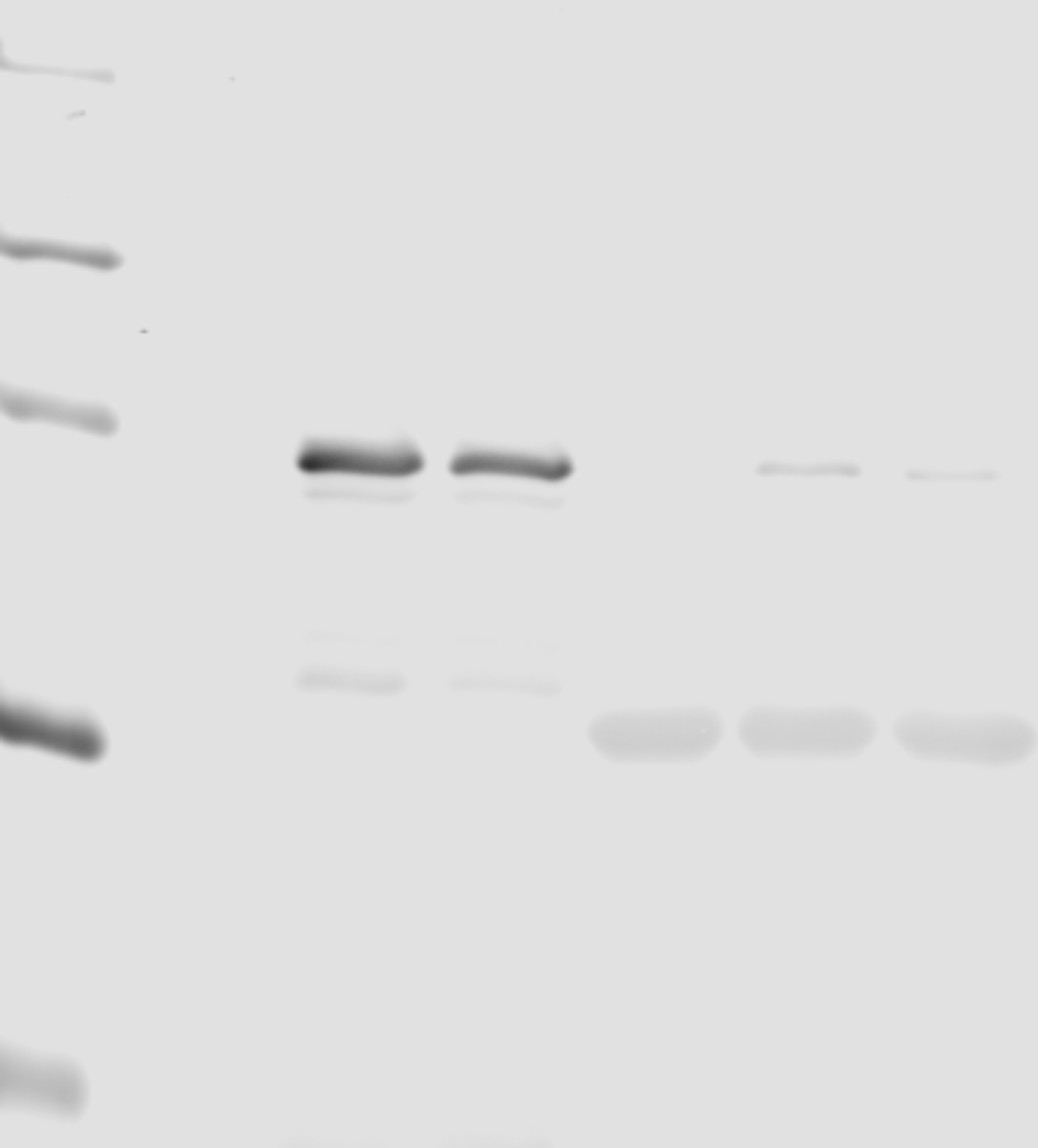

Supplement: Figure 2—source data 2. [file elife-91199-fig2-data2.zip › ib GFP.tif]

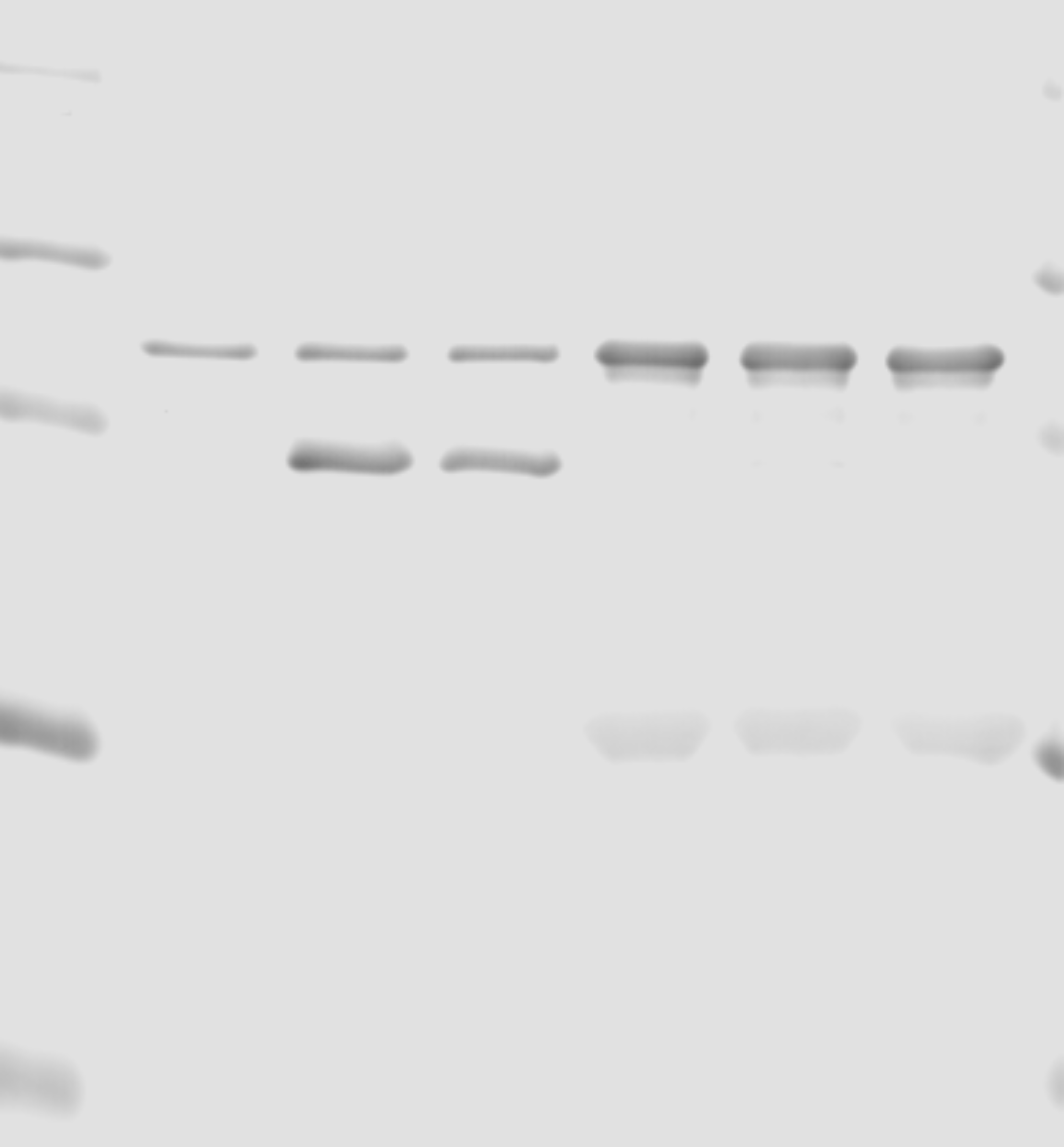

Supplement: Figure 2—source data 2. [file elife-91199-fig2-data2.zip › ib flag PK.tif]

Fig 2 Sup 1 a

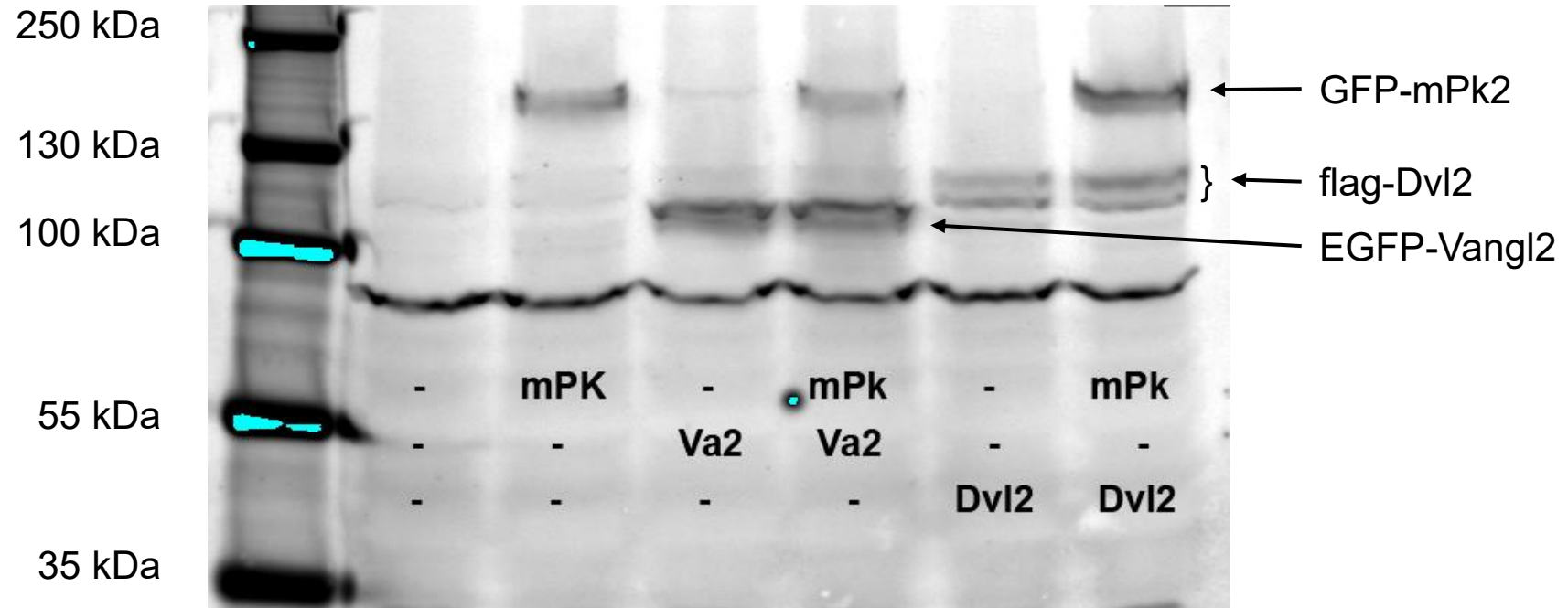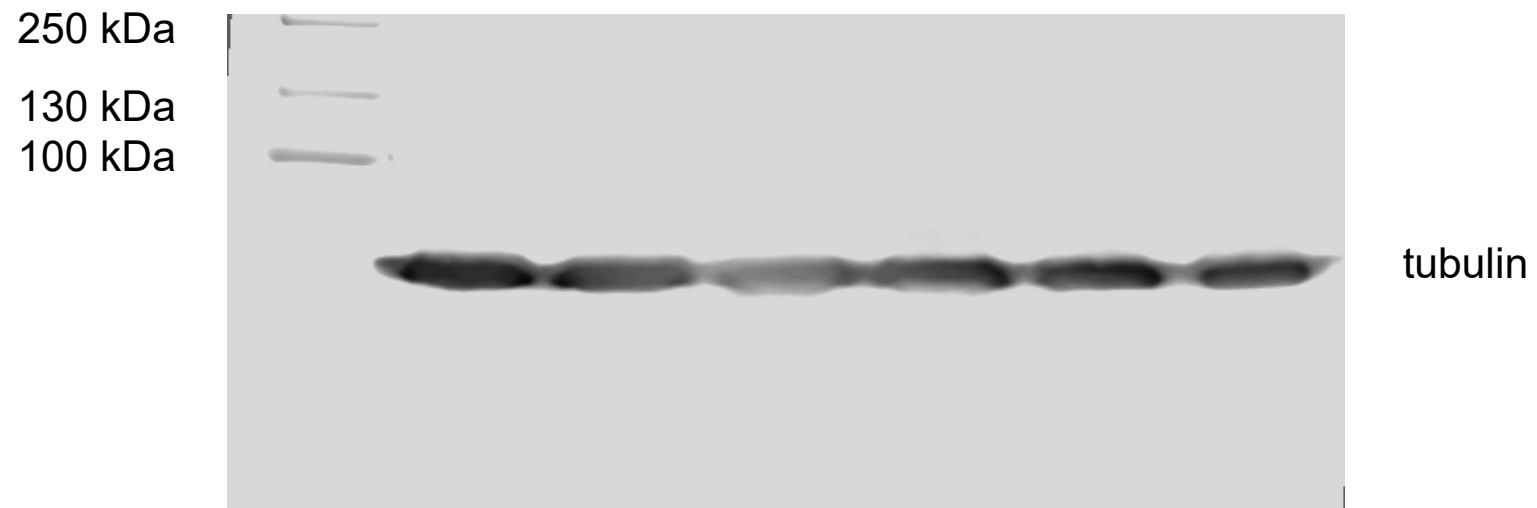

Fig 2 Sup 1 b

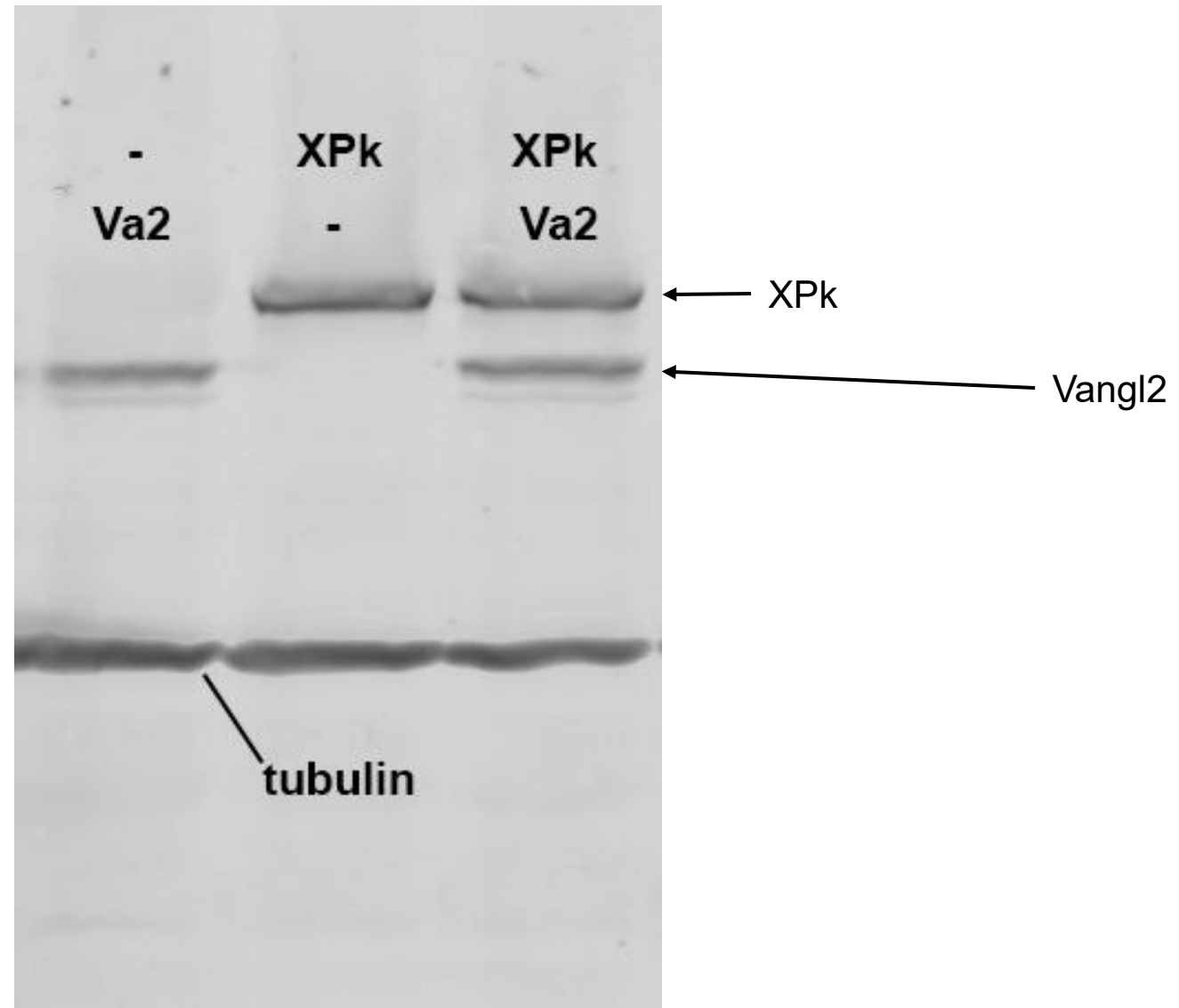

Fig 2 Sup 1 c

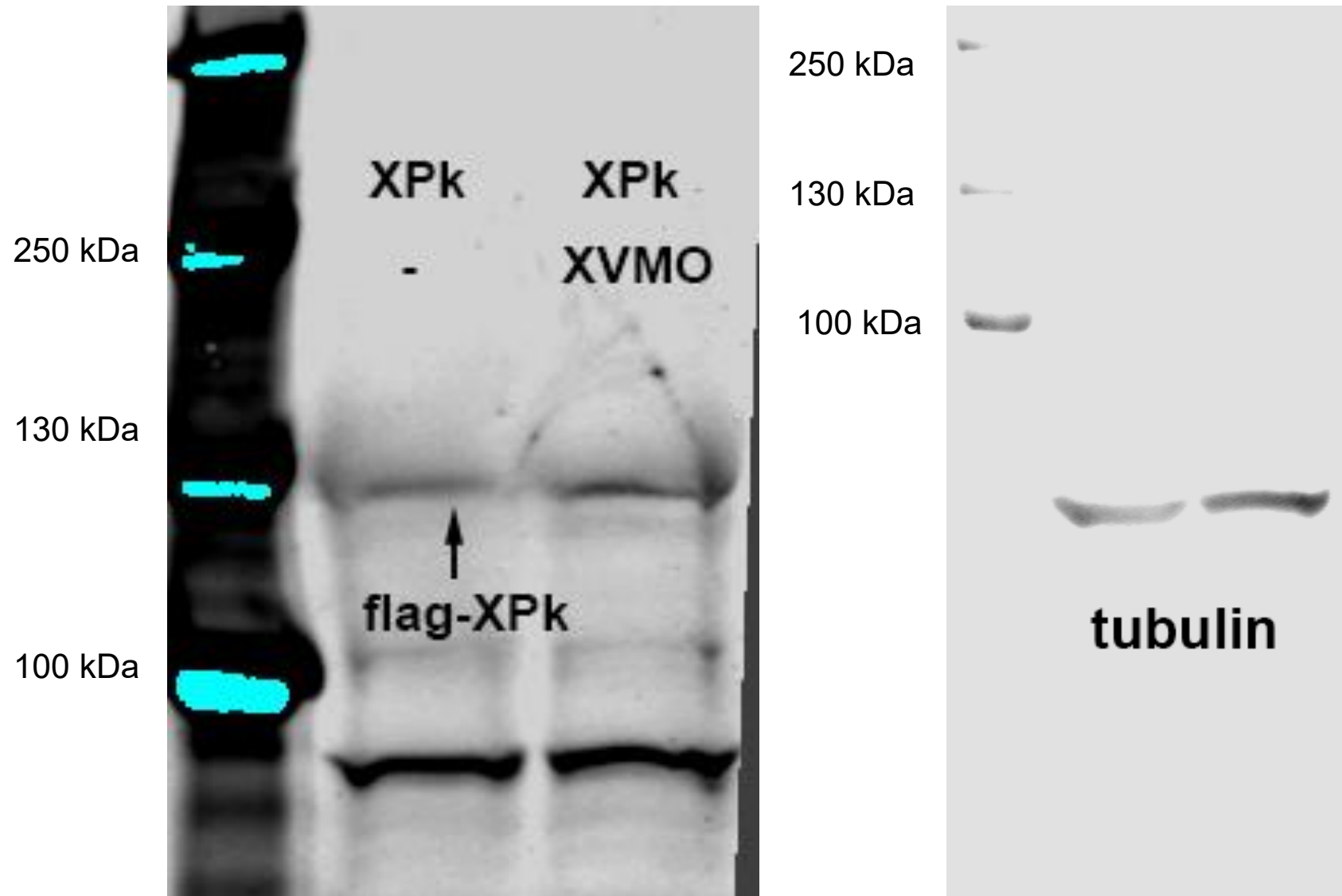

Fig 2 Sup 1 d

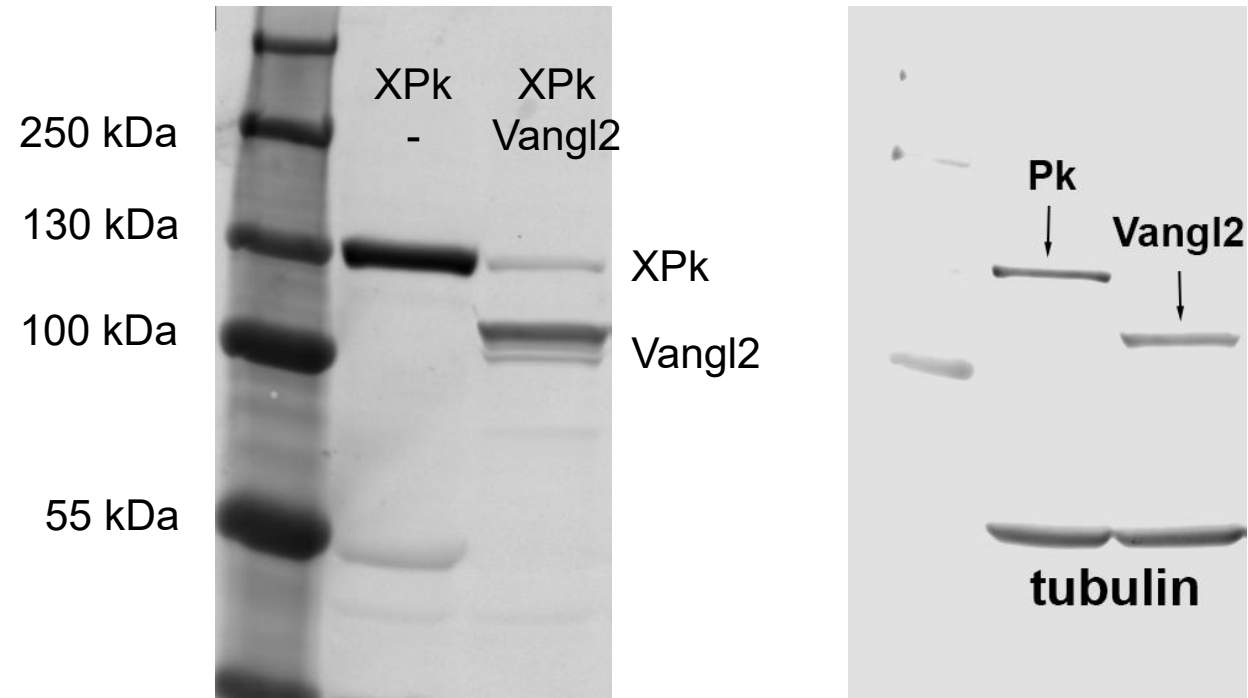

Supplement: Figure 2—figure supplement 1—source data 1. [file elife-91199-fig2-figsupp1-data1.zip › Figure 2 sup 1-source data_1.pdf]

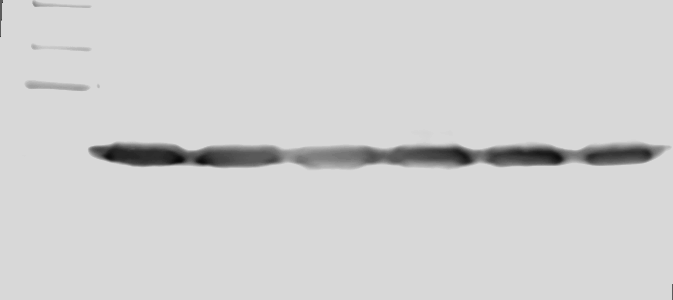

Supplement: Figure 2—figure supplement 1—source data 2. [file elife-91199-fig2-figsupp1-data2.zip › FIgure 2 sup 1-source data_2/Fig 2 sup 1a tubulin.tif]

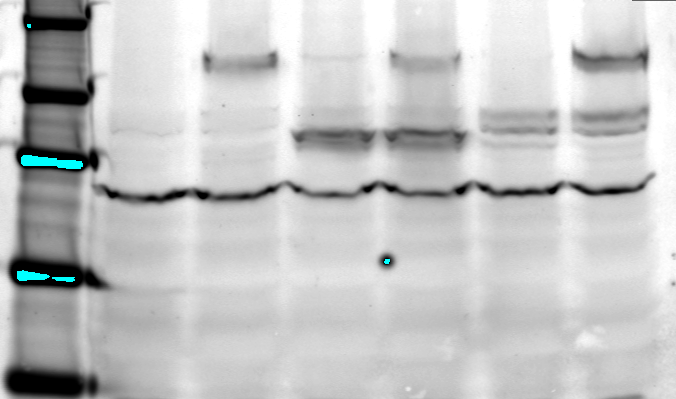

Supplement: Figure 2—figure supplement 1—source data 2. [file elife-91199-fig2-figsupp1-data2.zip › FIgure 2 sup 1-source data_2/Fig 2 sup 1a.tif]

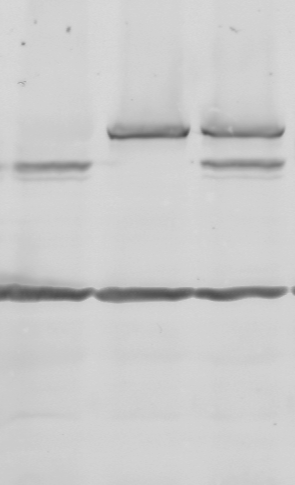

Supplement: Figure 2—figure supplement 1—source data 2. [file elife-91199-fig2-figsupp1-data2.zip › FIgure 2 sup 1-source data_2/FIg2 sup 1b.tif]

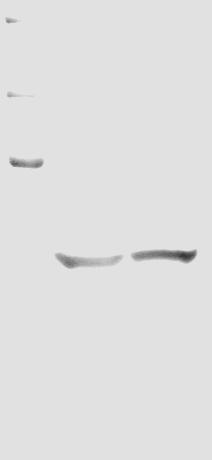

Supplement: Figure 2—figure supplement 1—source data 2. [file elife-91199-fig2-figsupp1-data2.zip › FIgure 2 sup 1-source data_2/Fig2 sup 1c tubulin.tif]

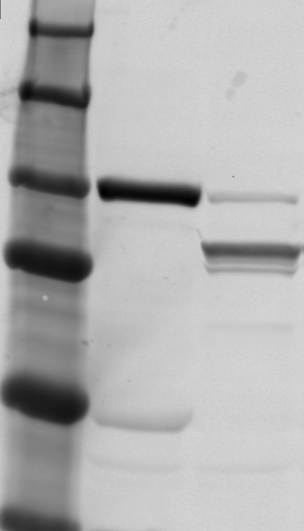

Supplement: Figure 2—figure supplement 1—source data 2. [file elife-91199-fig2-figsupp1-data2.zip › FIgure 2 sup 1-source data_2/Fig2 sup 1d .tif]

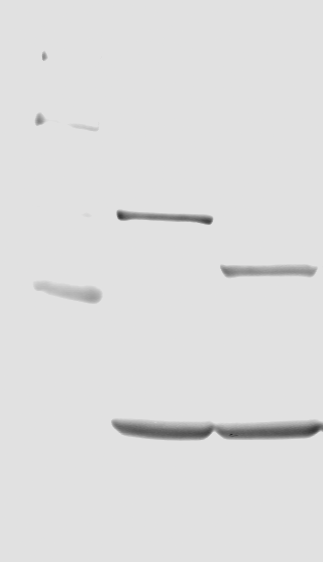

Supplement: Figure 2—figure supplement 1—source data 2. [file elife-91199-fig2-figsupp1-data2.zip › FIgure 2 sup 1-source data_2/Fig2 sup 1d tubulin.tif]

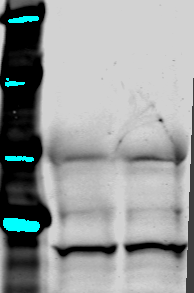

Supplement: Figure 2—figure supplement 1—source data 2. [file elife-91199-fig2-figsupp1-data2.zip › FIgure 2 sup 1-source data_2/Fig2 sup1c XPk.tif]

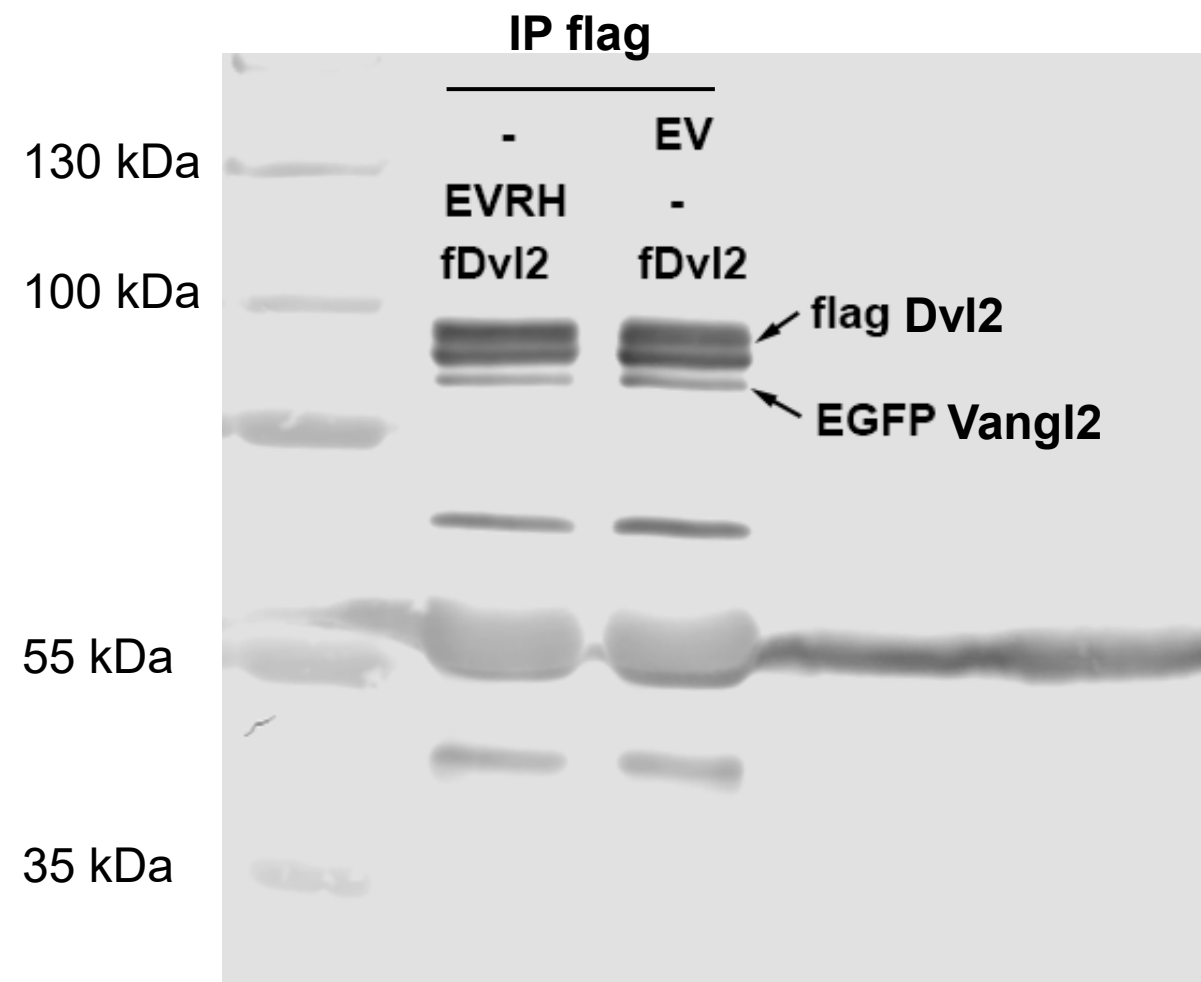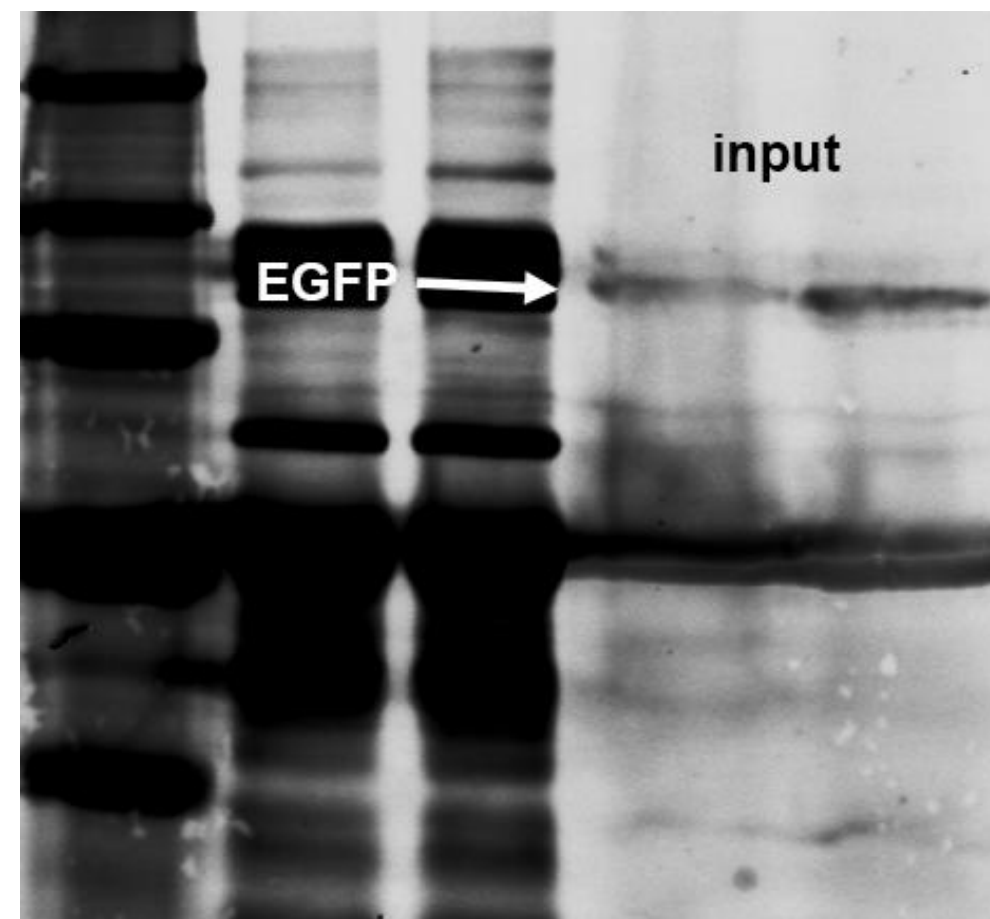

Supplement: Figure 2—figure supplement 4—source data 1. [file elife-91199-fig2-figsupp4-data1.zip › Figure 2 sup 4e- source data 1.pdf]

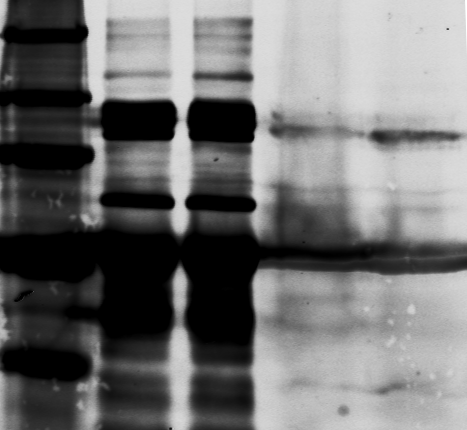

Supplement: Figure 2—figure supplement 4—source data 2. [file elife-91199-fig2-figsupp4-data2.zip › Figure 2 sup 4e-source data 2/EVfD_EVRHfD input GFP.tif]

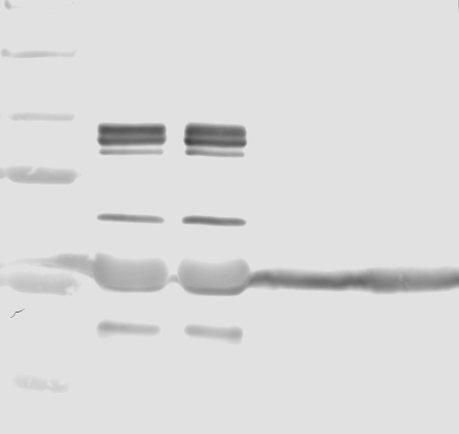

Supplement: Figure 2—figure supplement 4—source data 2. [file elife-91199-fig2-figsupp4-data2.zip › Figure 2 sup 4e-source data 2/EVfD_EVRHfD IP flag WB GFP.tif]

EGFP-Vangl2

+ + + + - + + + + -

Flag-Dvl2

+ + + + - + + + + -

xWnt11

- + - + - - + - + -

mPK2-GFP

- - + + - - - + + -

130 kDa

input

Co-IP

100 kDa

EGFP-Vangl2

55 kDa

35 kDa

IB Vangl2

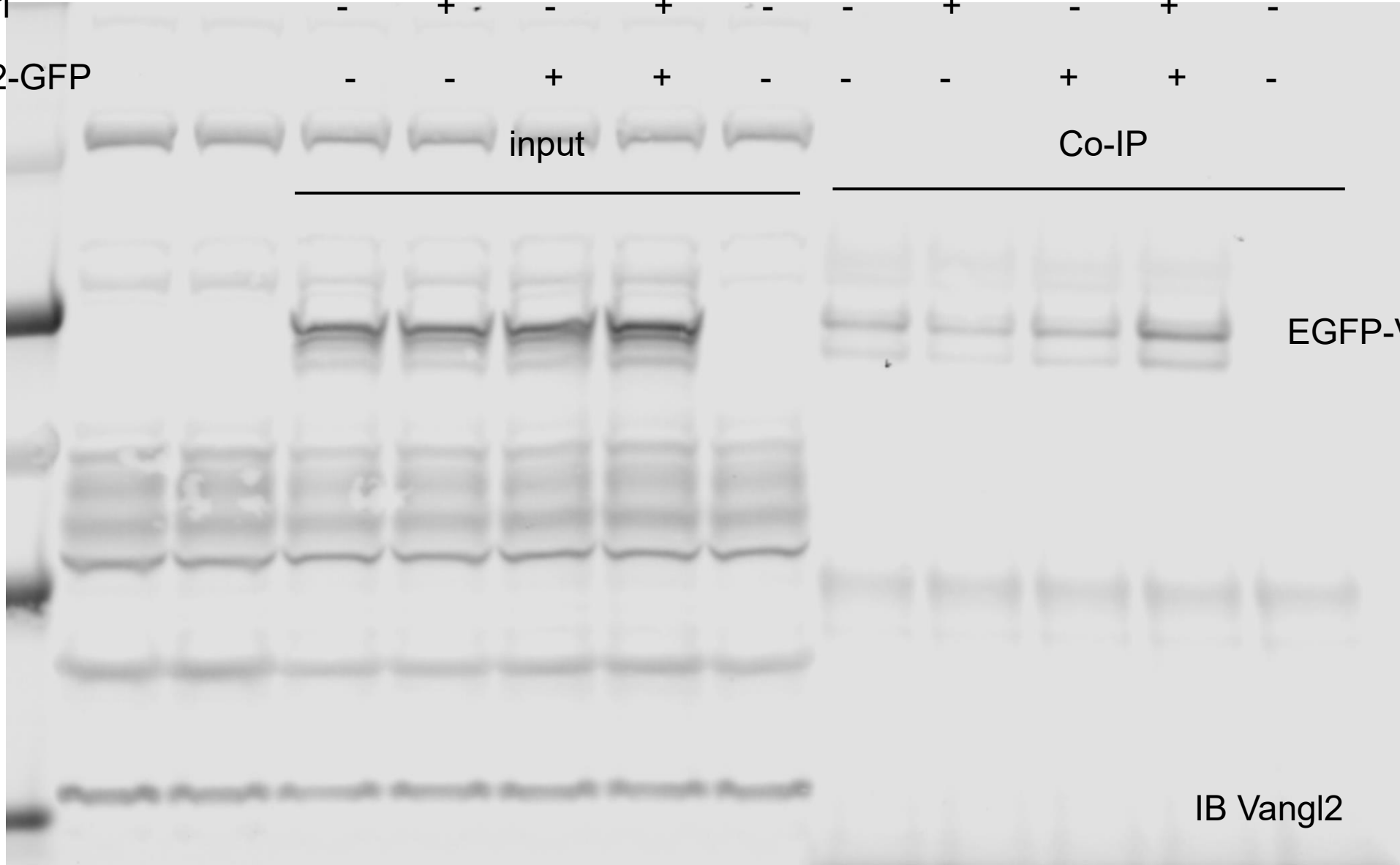

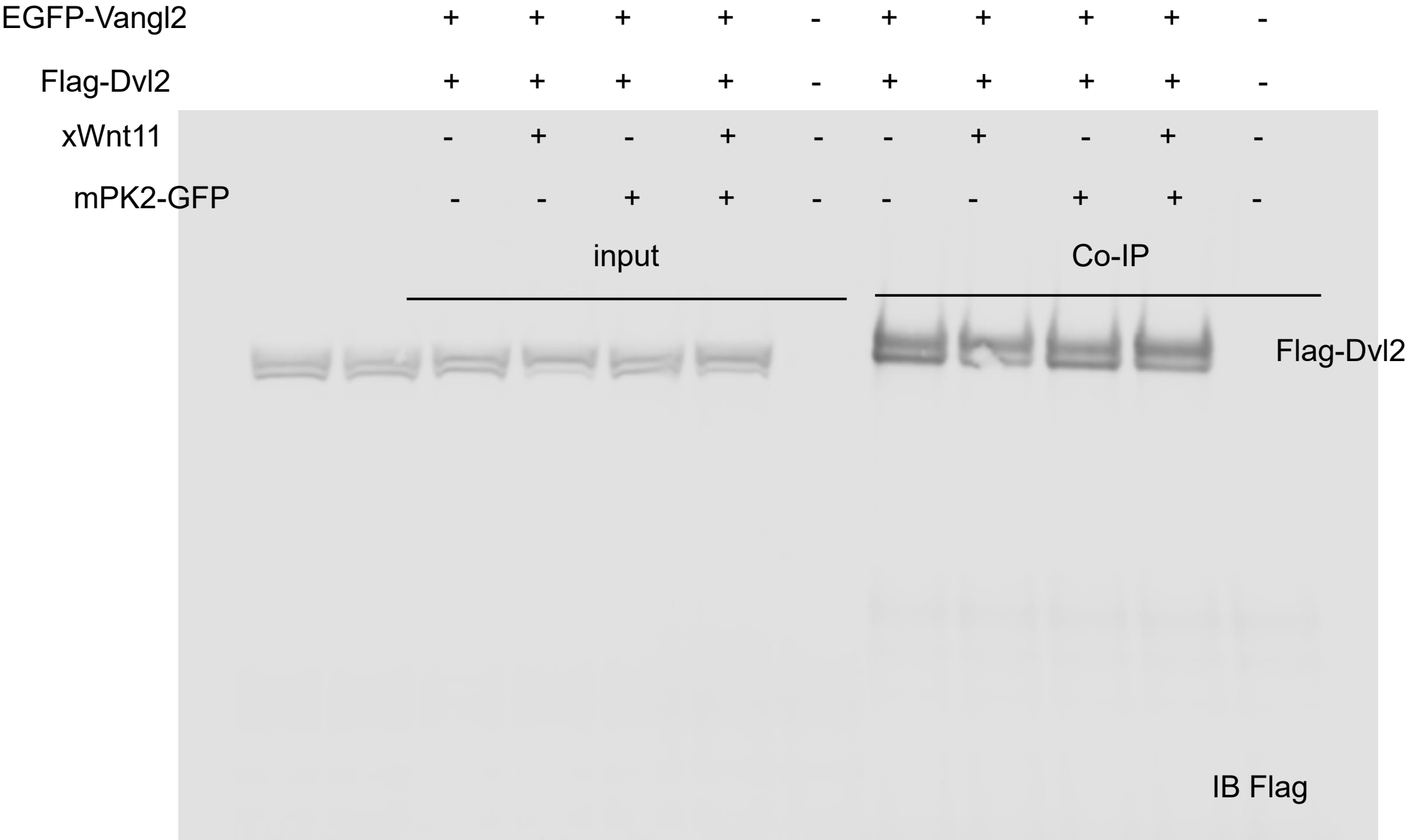

Supplement: Figure 3—figure supplement 2—source data 1. [file elife-91199-fig3-figsupp2-data1.zip › Figure 3 suppl 2a- source data 1.pdf]

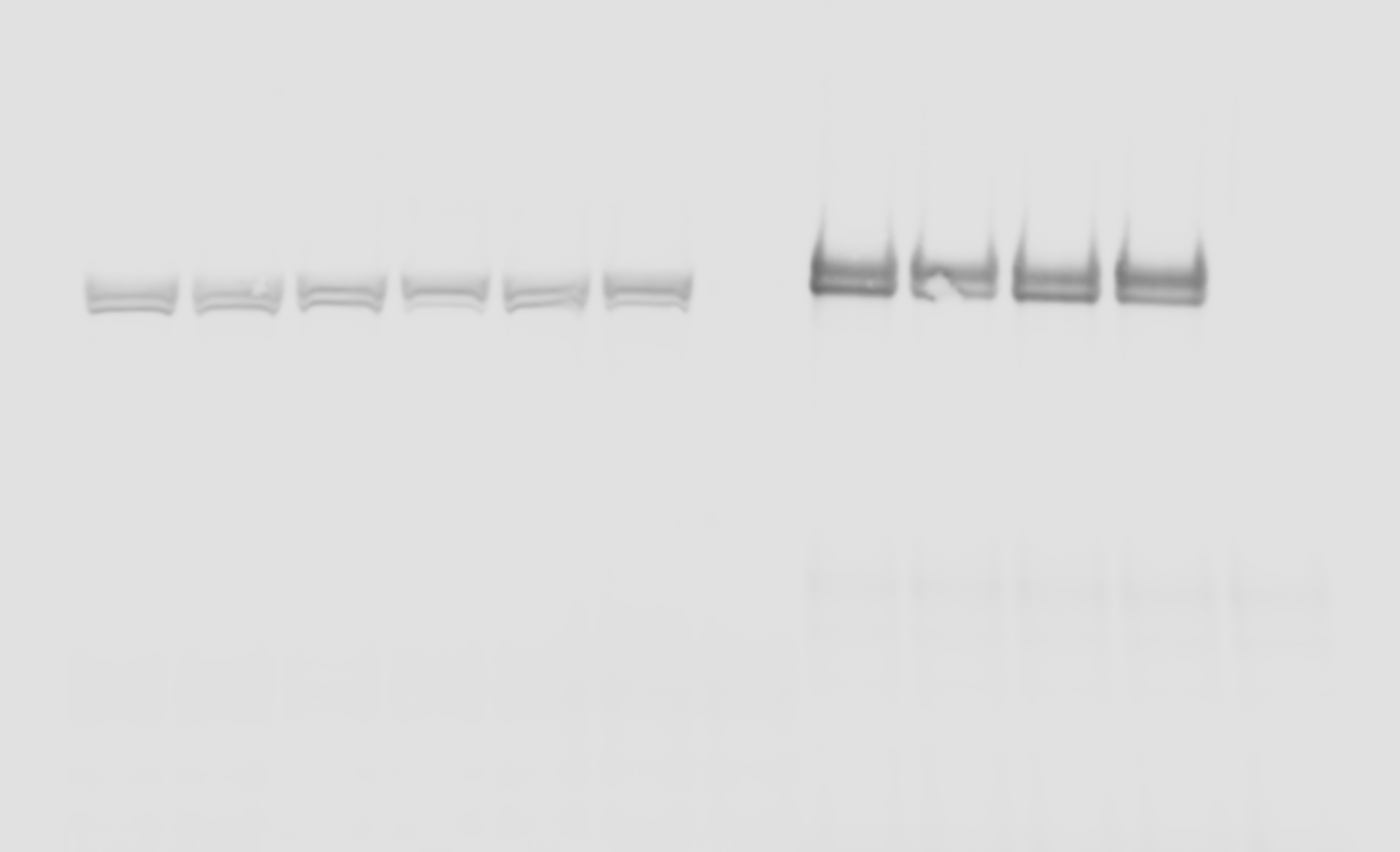

Supplement: Figure 3—figure supplement 2—source data 2. [file elife-91199-fig3-figsupp2-data2.zip › 1.7 inj, ib Flag, Co-IP DV add PK and Wnt11.tif]

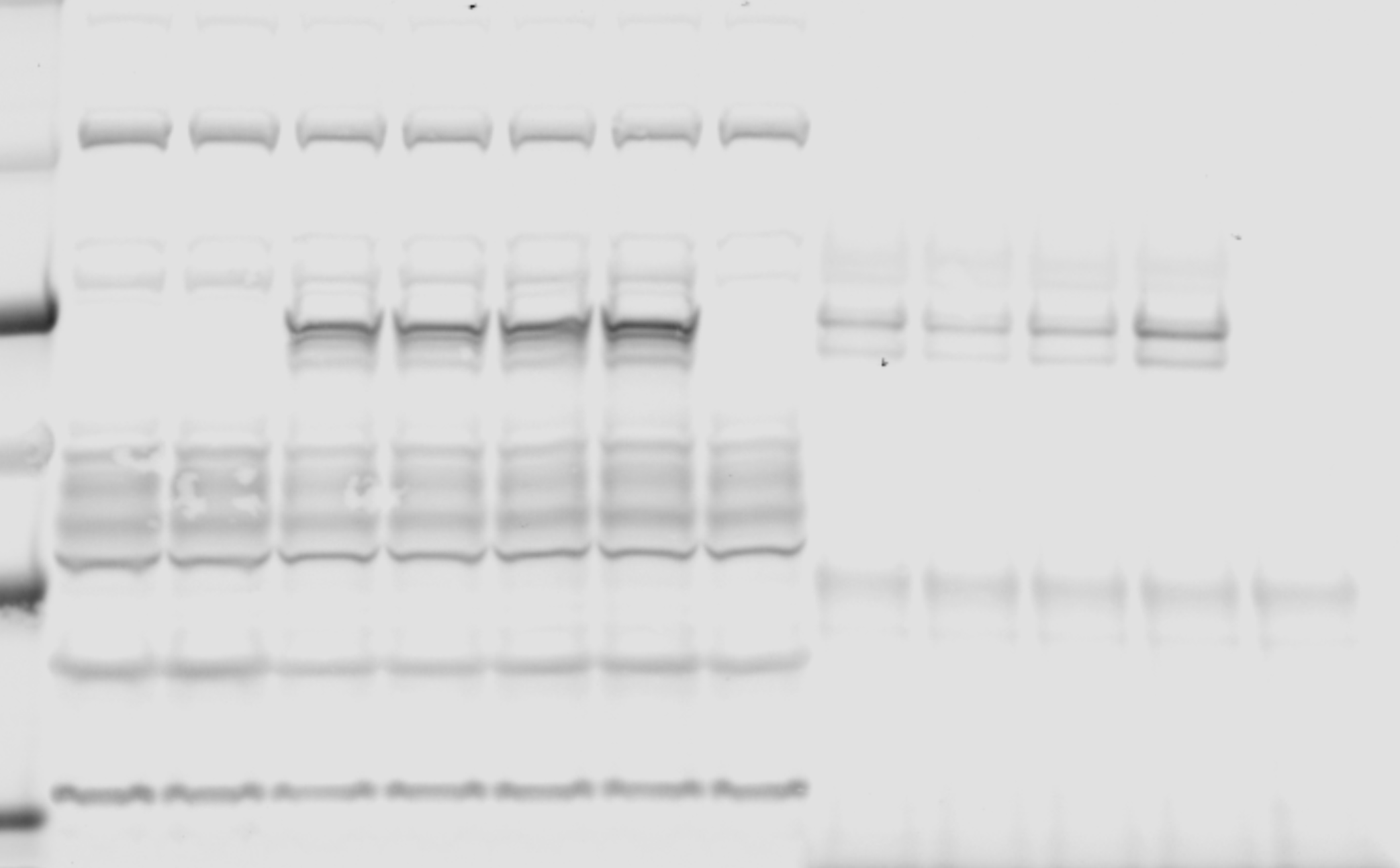

Supplement: Figure 3—figure supplement 2—source data 2. [file elife-91199-fig3-figsupp2-data2.zip › 1.7 inj ib Vangl2, DV Co-IP add Pk and Wnt11.tif]

Fig 6a

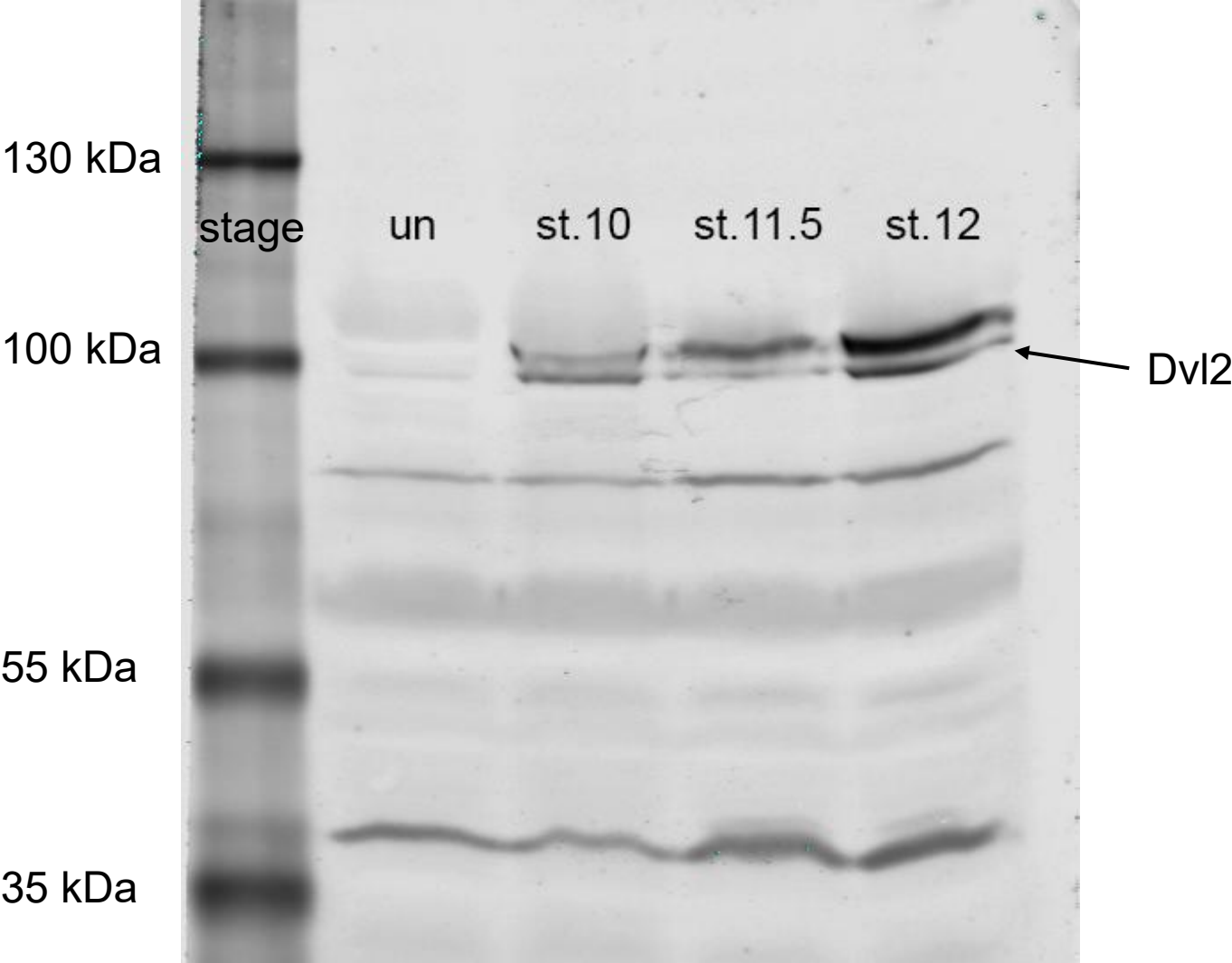

Fig 6b

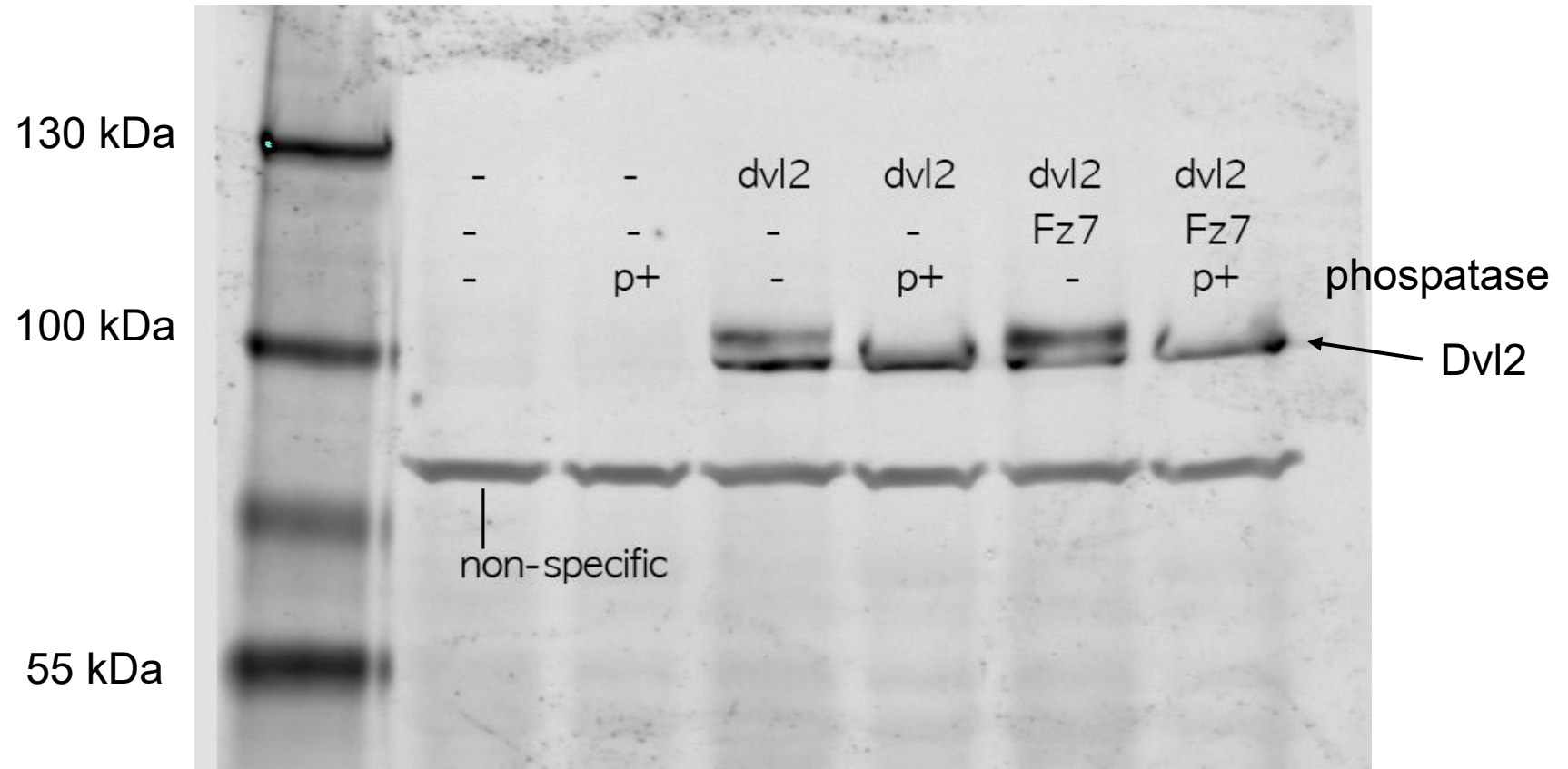

Fig 6c

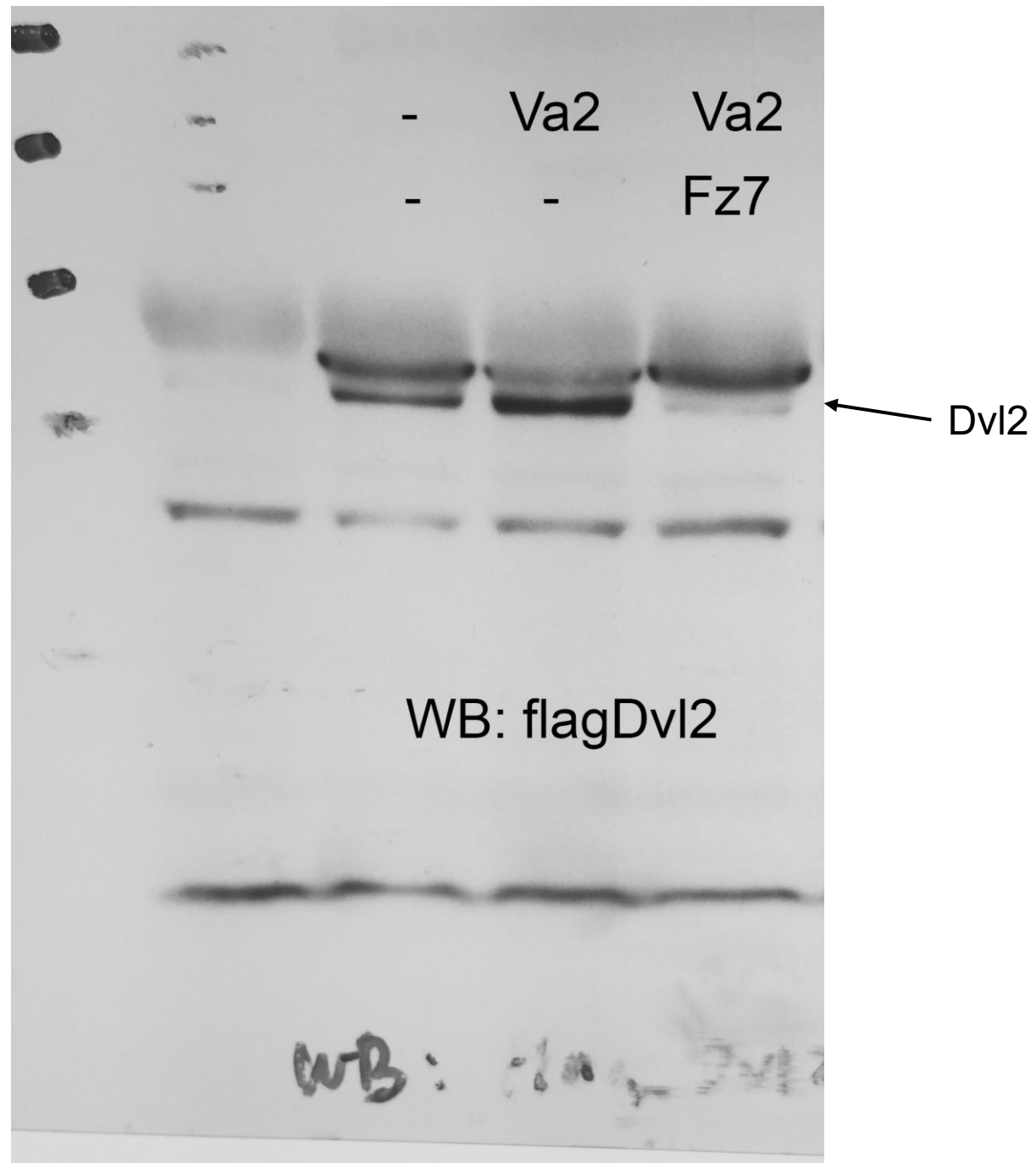

Fig 6d

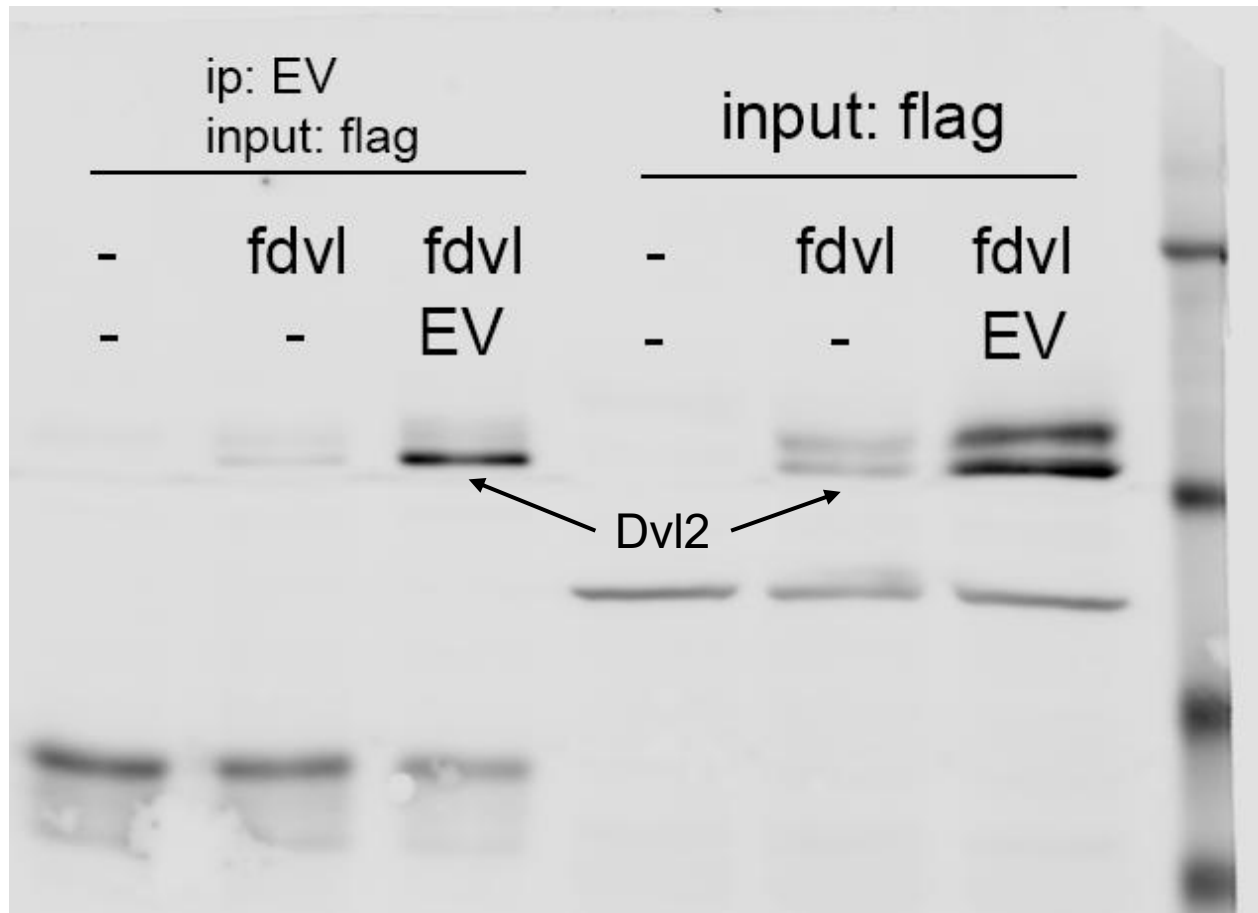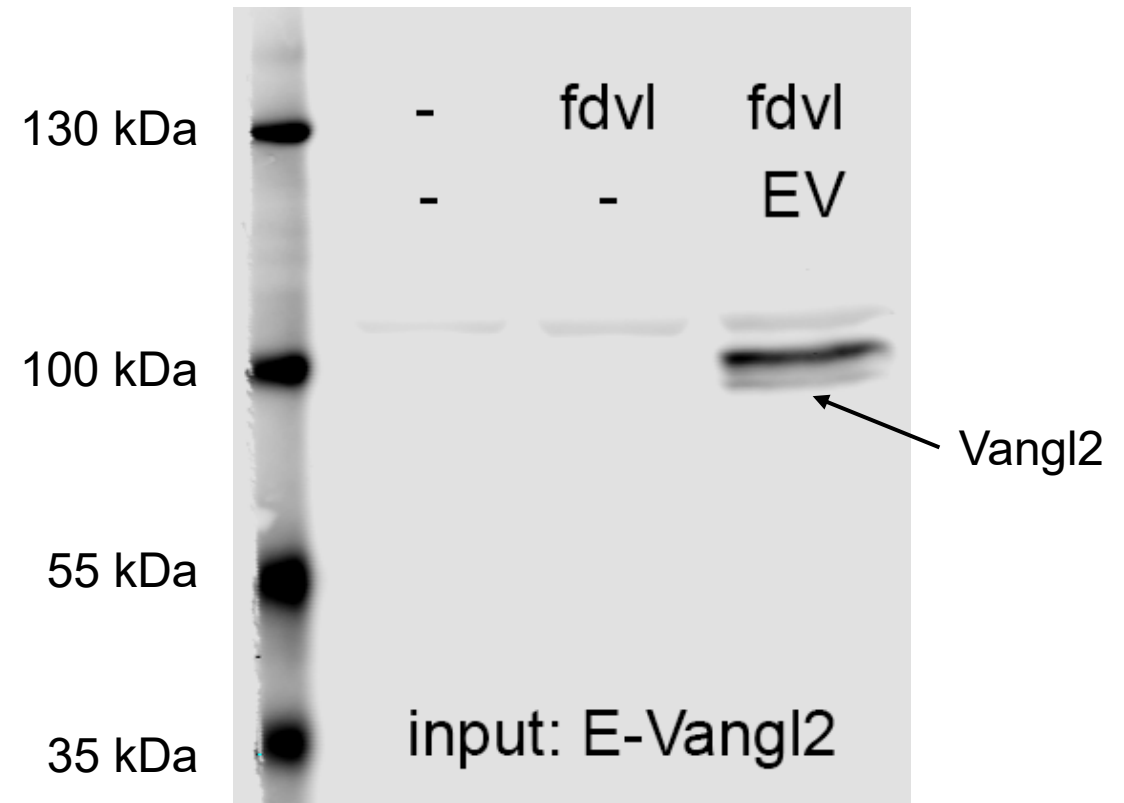

Fig 6e

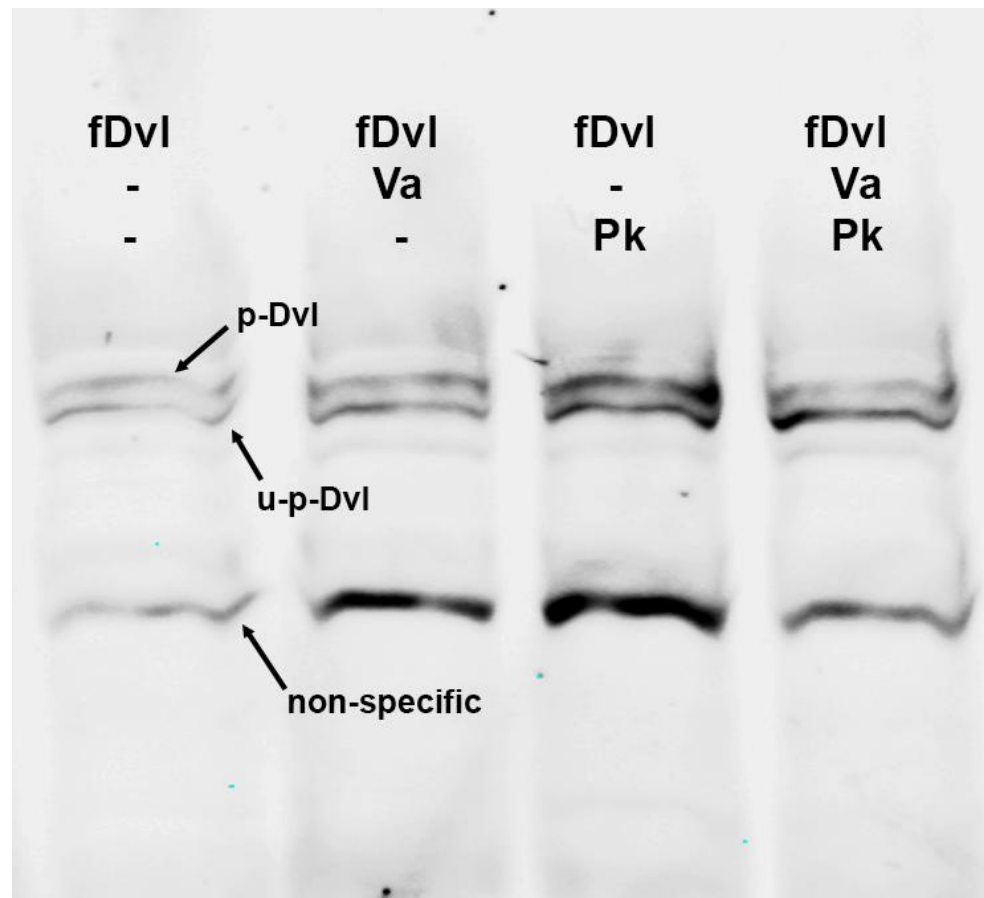

Supplement: Figure 6—source data 1. [file elife-91199-fig6-data1.zip › Figure 6-source data 1 .pdf]

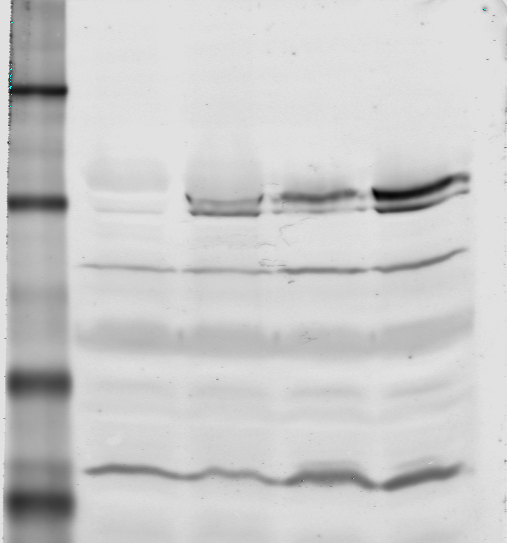

Supplement: Figure 6—source data 2. [file elife-91199-fig6-data2.zip › FIgure 6-source data 2/Fig 6a. un-10-11.5-12_.tif]

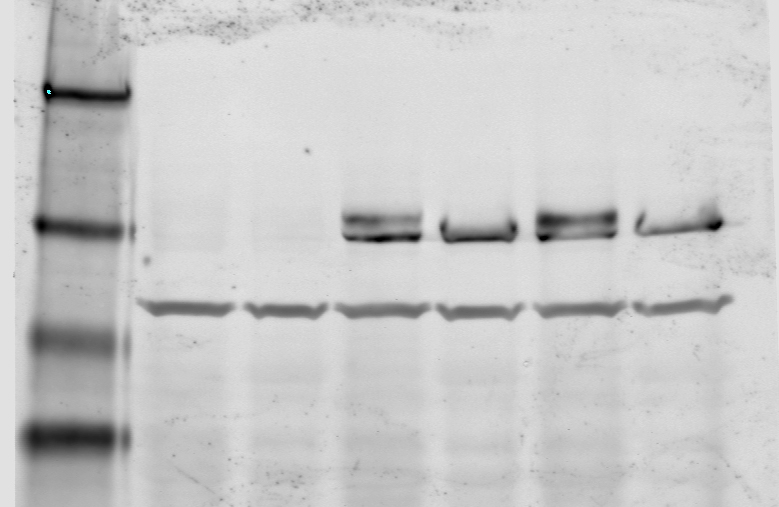

Supplement: Figure 6—source data 2. [file elife-91199-fig6-data2.zip › FIgure 6-source data 2/Fig 6b phosphatase - uninjected_dvl_dvl-fz_.tif]

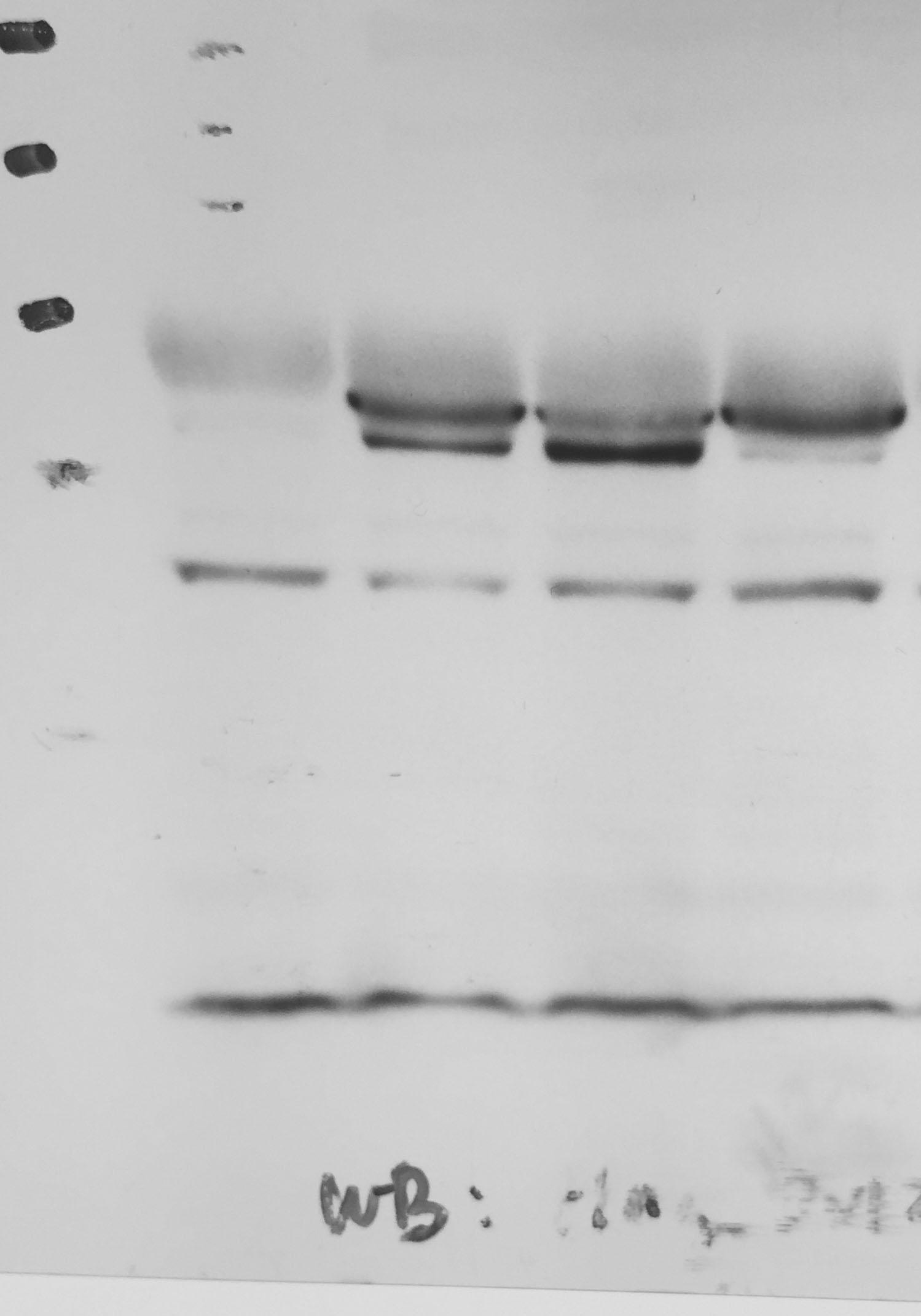

Supplement: Figure 6—source data 2. [file elife-91199-fig6-data2.zip › FIgure 6-source data 2/Fig 6c .jpg]

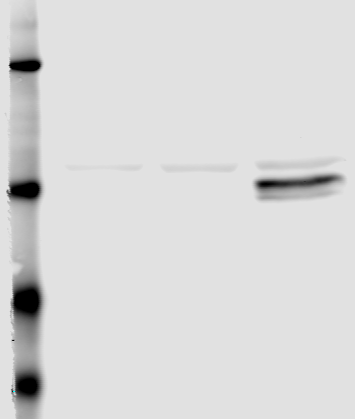

Supplement: Figure 6—source data 2. [file elife-91199-fig6-data2.zip › FIgure 6-source data 2/Fig 6d Dvl2-Vangl2_input-Vangl2.tif]

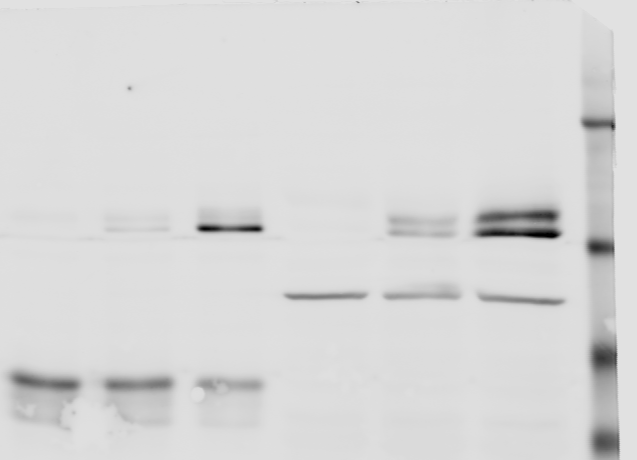

Supplement: Figure 6—source data 2. [file elife-91199-fig6-data2.zip › FIgure 6-source data 2/Fig 6d Dvl2-Vangl2_IP-Vangl2_WB-Dvl2.tif]

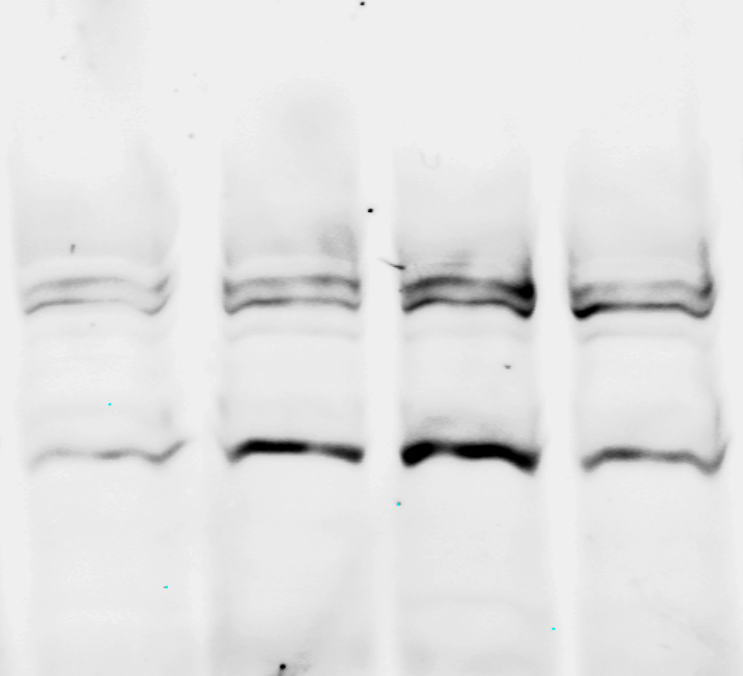

Supplement: Figure 6—source data 2. [file elife-91199-fig6-data2.zip › FIgure 6-source data 2/Fig 6e fDvl_fDvl EV_fDvl Pk_fDvl EV Pk_fDvl WB flag 02.tif]

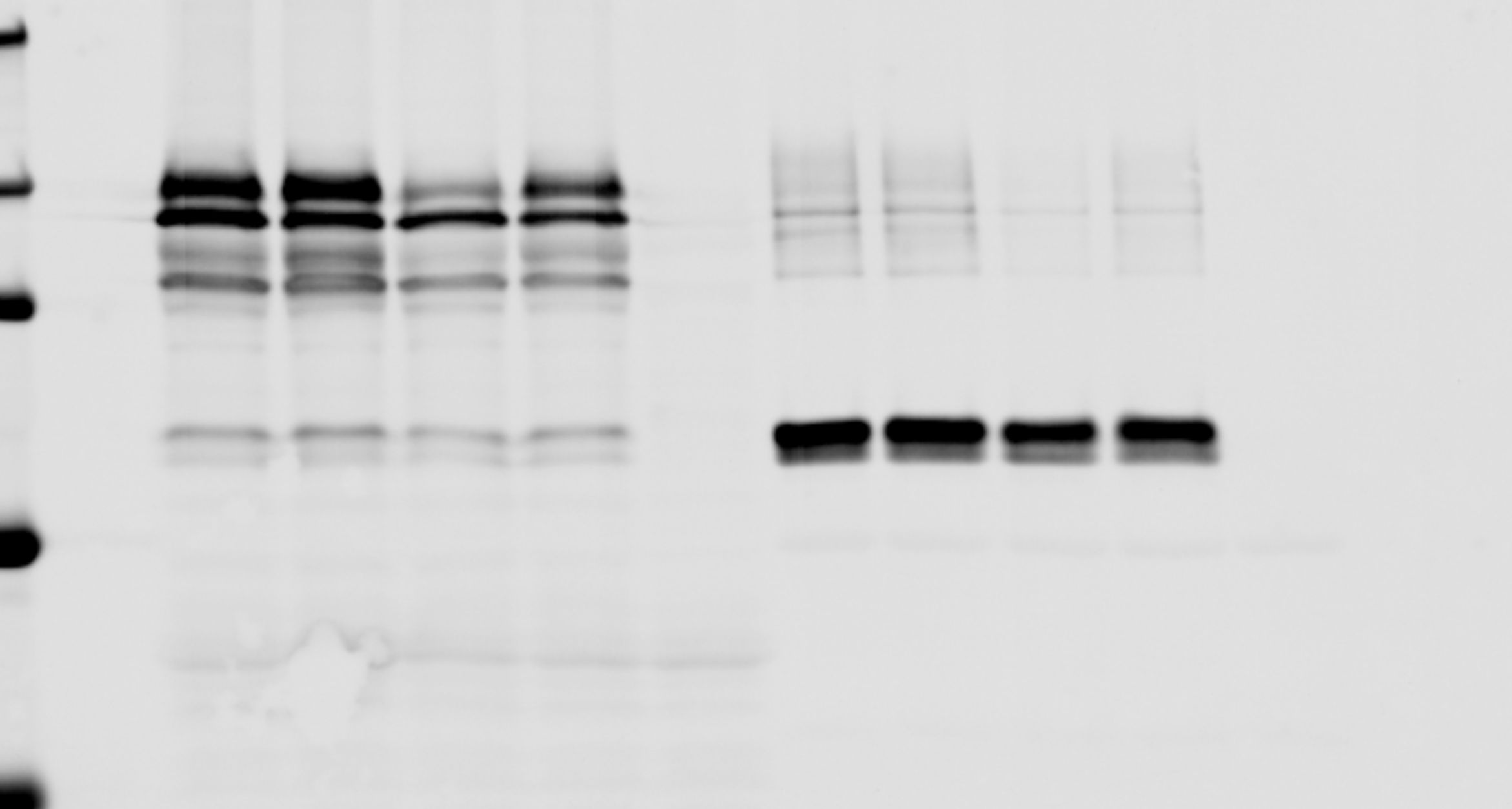

Supplement: Figure 7—figure supplement 2—source data 2. [file elife-91199-fig7-figsupp2-data2.zip › Figure 7 sup 2-source data 2/Fig 7 sup 2a.tif]

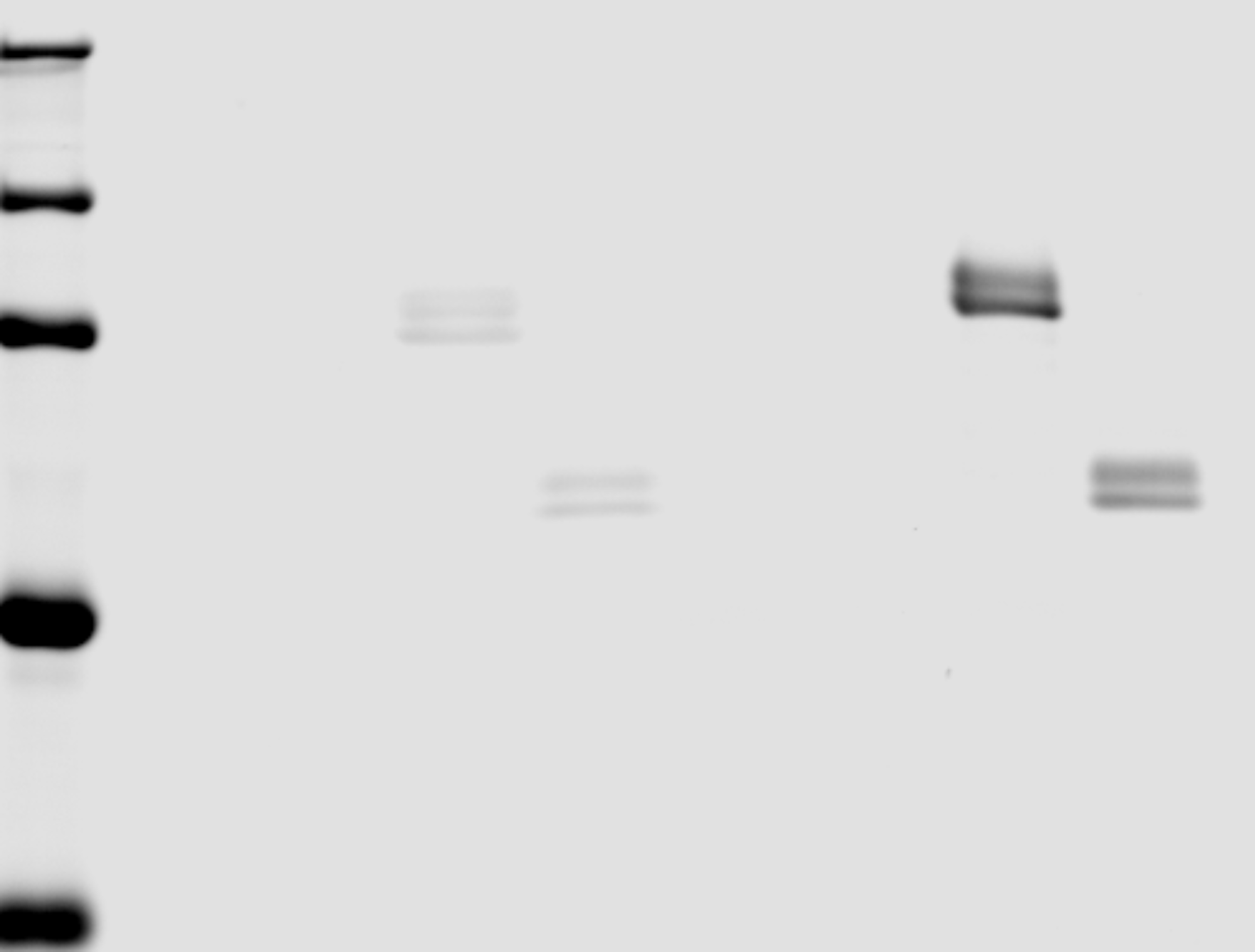

Supplement: Figure 7—figure supplement 2—source data 2. [file elife-91199-fig7-figsupp2-data2.zip › Figure 7 sup 2-source data 2/Fig 7 sup 2b ib myc.tif]

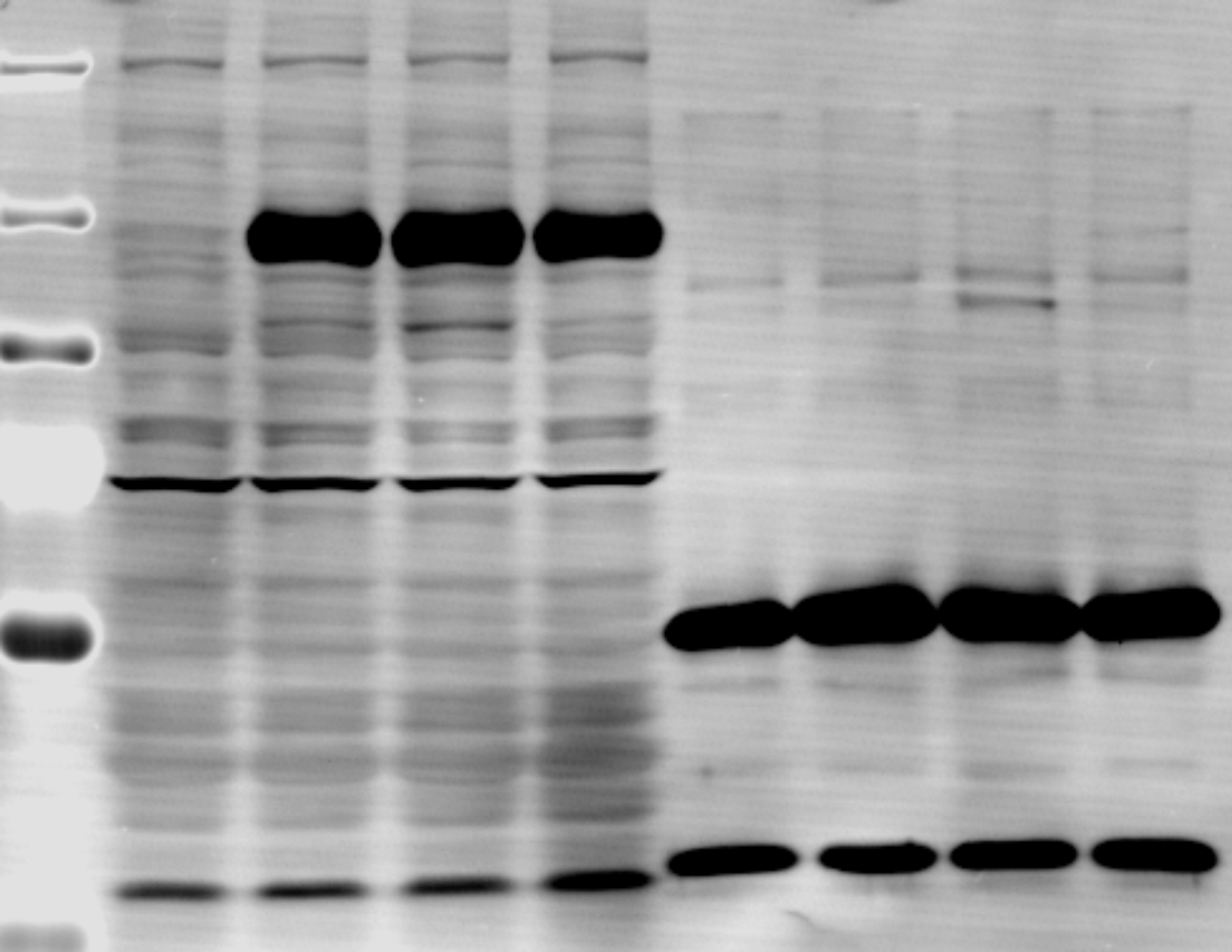

Supplement: Figure 7—figure supplement 2—source data 2. [file elife-91199-fig7-figsupp2-data2.zip › Figure 7 sup 2-source data 2/Fig 7 sup 2b ib Ror2.tif]

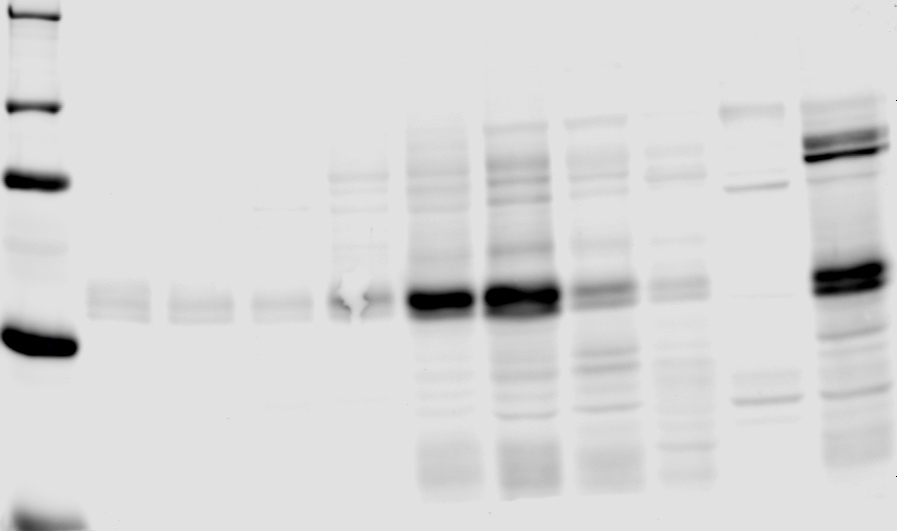

Supplement: Figure 7—figure supplement 2—source data 2. [file elife-91199-fig7-figsupp2-data2.zip › Figure 7 sup 2-source data 2/Fig 7 sup 2c _Dvl.tif]

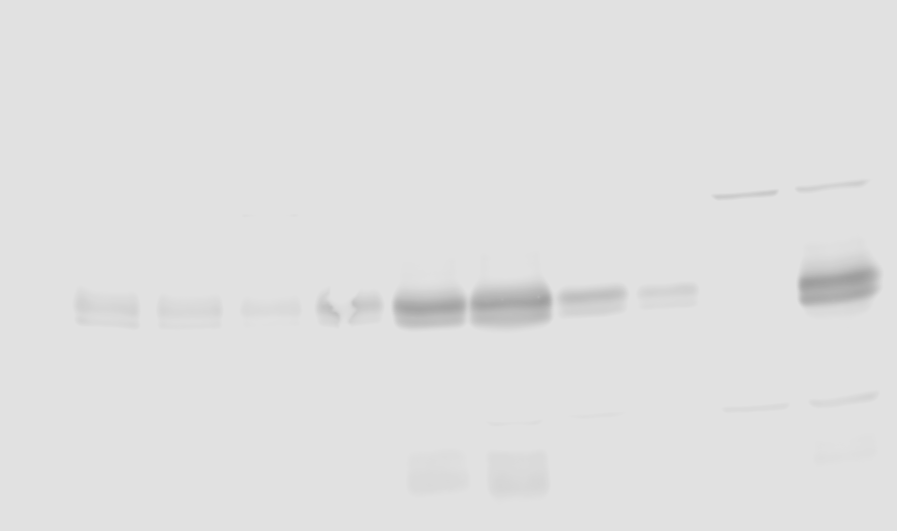

Supplement: Figure 7—figure supplement 2—source data 2. [file elife-91199-fig7-figsupp2-data2.zip › Figure 7 sup 2-source data 2/Fig 7 sup 2c_HA-Vangl2.tif]

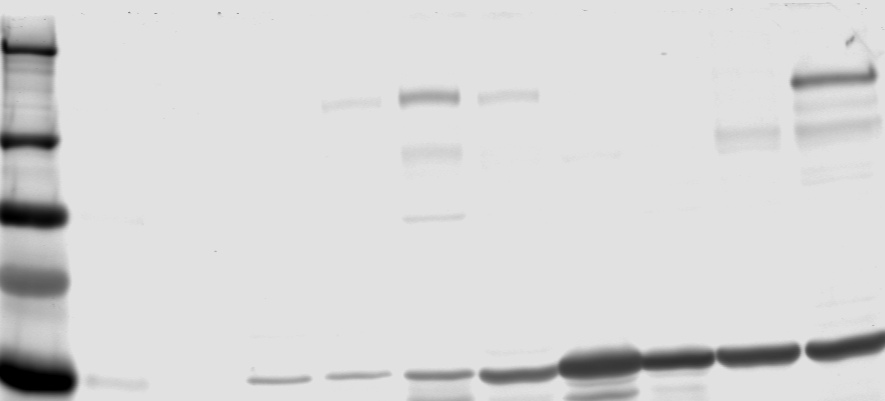

Supplement: Figure 7—figure supplement 2—source data 2. [file elife-91199-fig7-figsupp2-data2.zip › Figure 7 sup 2-source data 2/Fig 7 sup 2c_Ror2.tif]
