# Supplementary material for: Prickle and Ror modulate Dishevelled-Vangl interaction to regulate non-canonical Wnt signaling during convergent extension in Xenopus
Source: eLife. 2026 Apr 30;12:RP91199. doi: 10.7554/eLife.91199 (PMC13132548; doi:10.7554/eLife.91199)
Supplement: Figure 7—figure supplement 2—source data 1. [file elife-91199-fig7-figsupp2-data1.zip › Figure 7 sup 2-source data 1.pdf]

Fig 7 sup 2a High-exposure

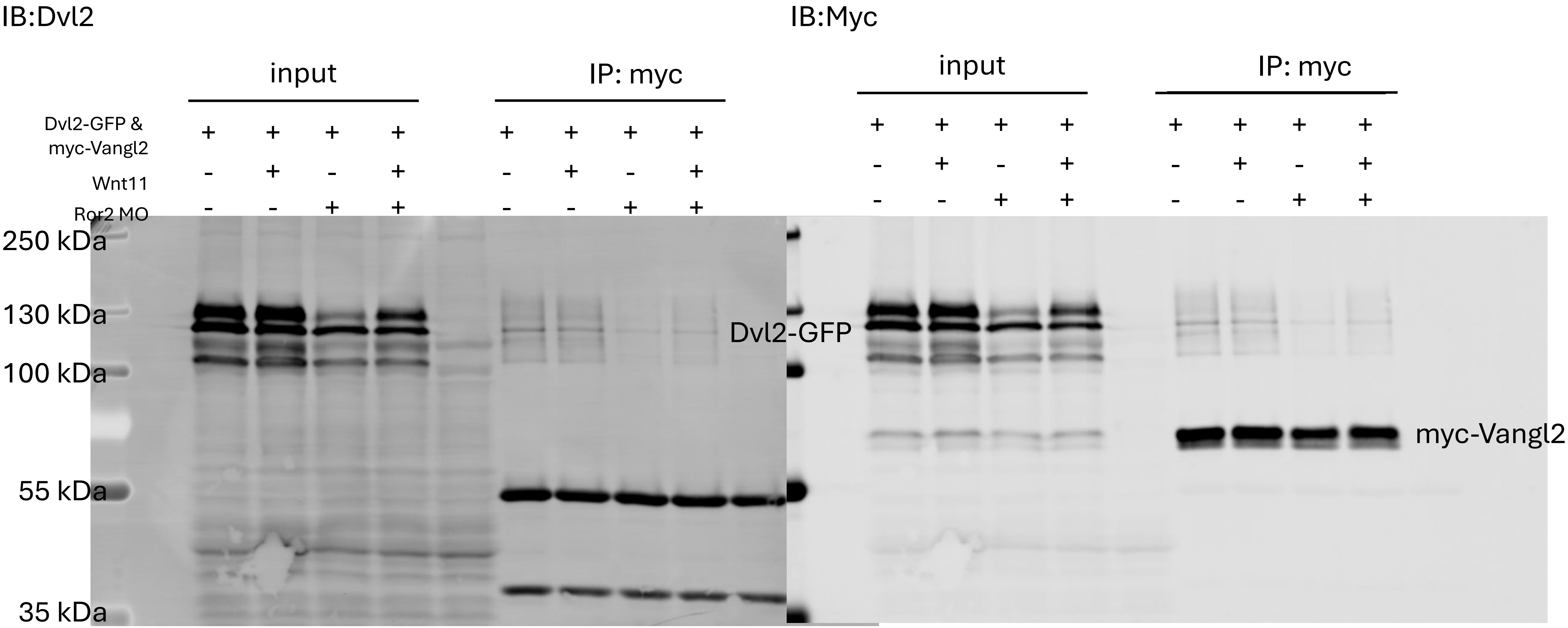

Fig 7 sup 2a   low-exposure

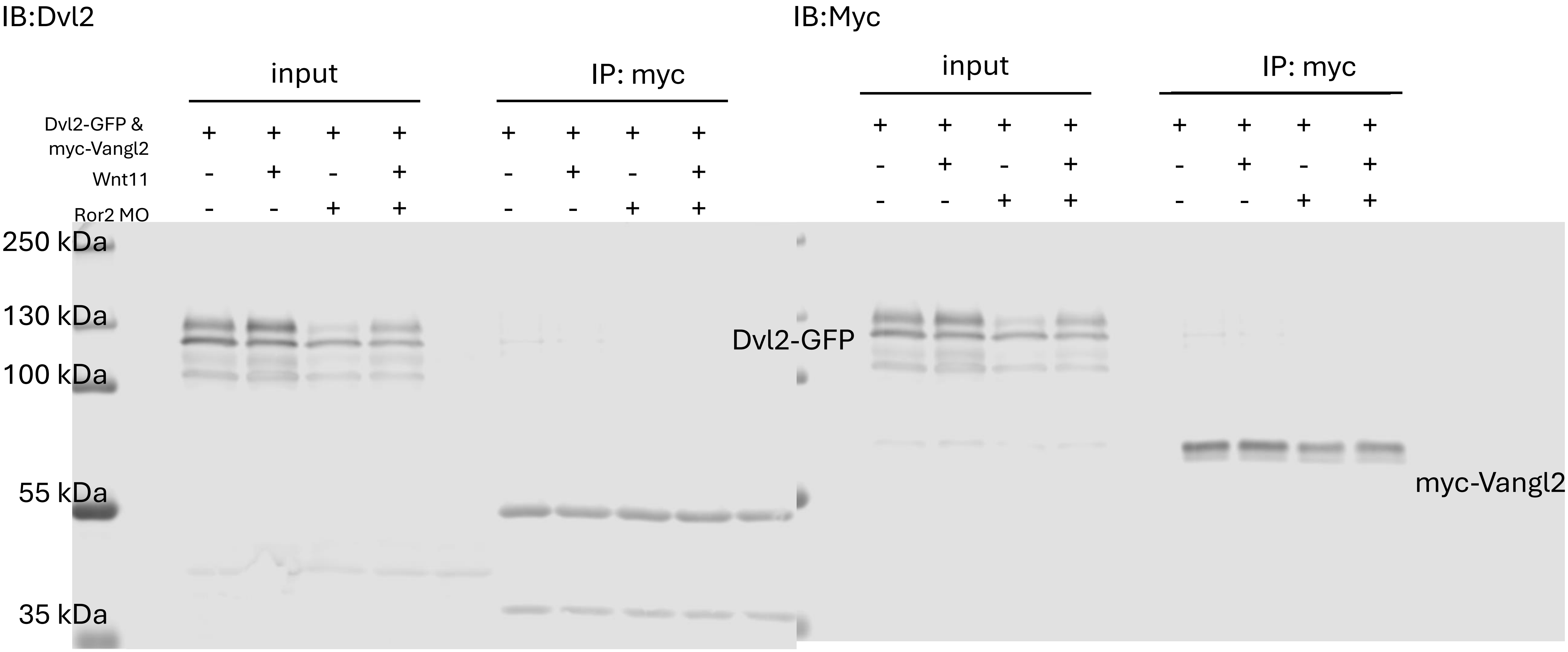

Fig 7 sup 2b

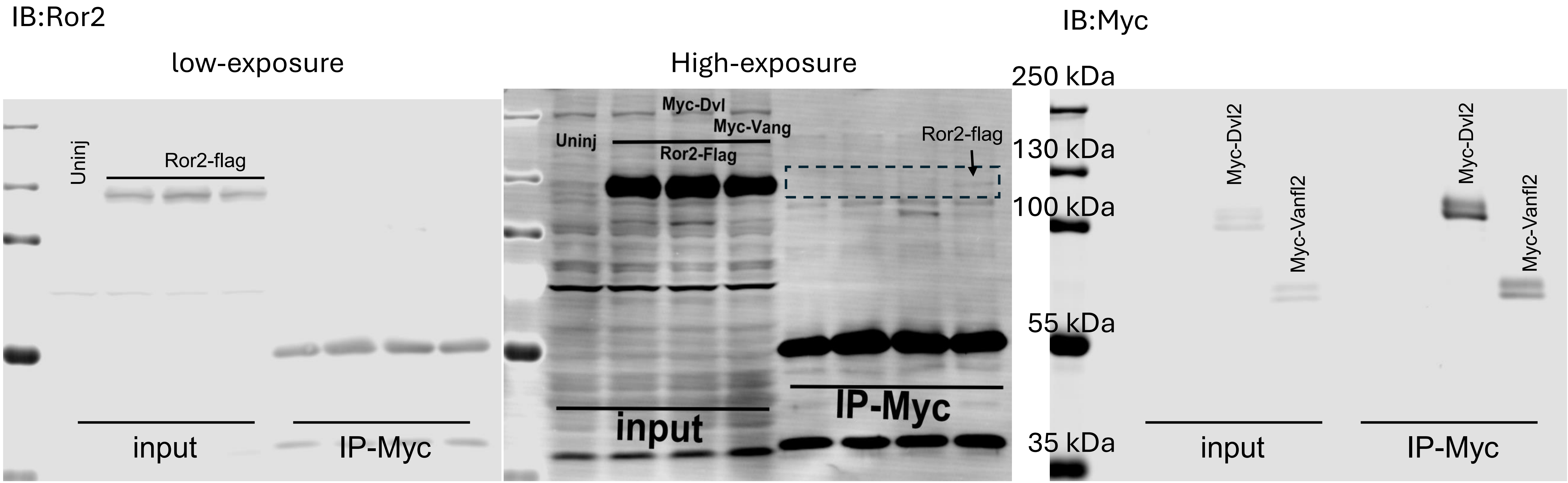

IB:Ror2
low-exposure
Uninj
Ror2-flag
input
IP-Myc
High-exposure
Myc-Dvl
Myc-Vang
Uninj
Ror2-Flag
Ror2-flag
input
IP-Myc
IB:Myc
250 kDa
130 kDa
100 kDa
55 kDa
35 kDa
Myc-Dvl2
Myc-Vanfl2
Myc-Dvl2
Myc-Vanfl2
input
IP-Myc

Fig 7 sup 2c

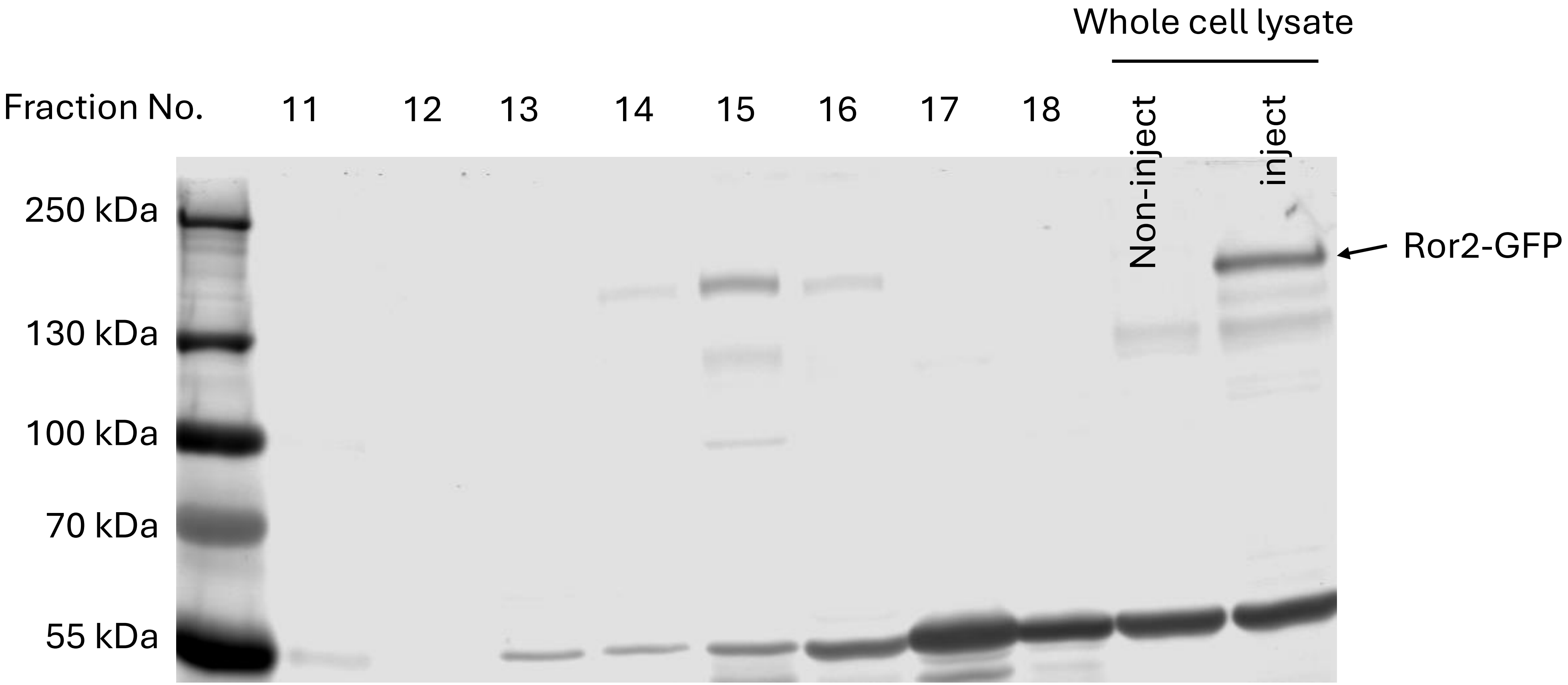
Whole cell lysate
Fraction No.
11
12
13
14
15
16
17
18
Non-inject
inject
250 kDa
130 kDa
100 kDa
70 kDa
55 kDa
Ror2-GFP

Fig 7 sup 2c

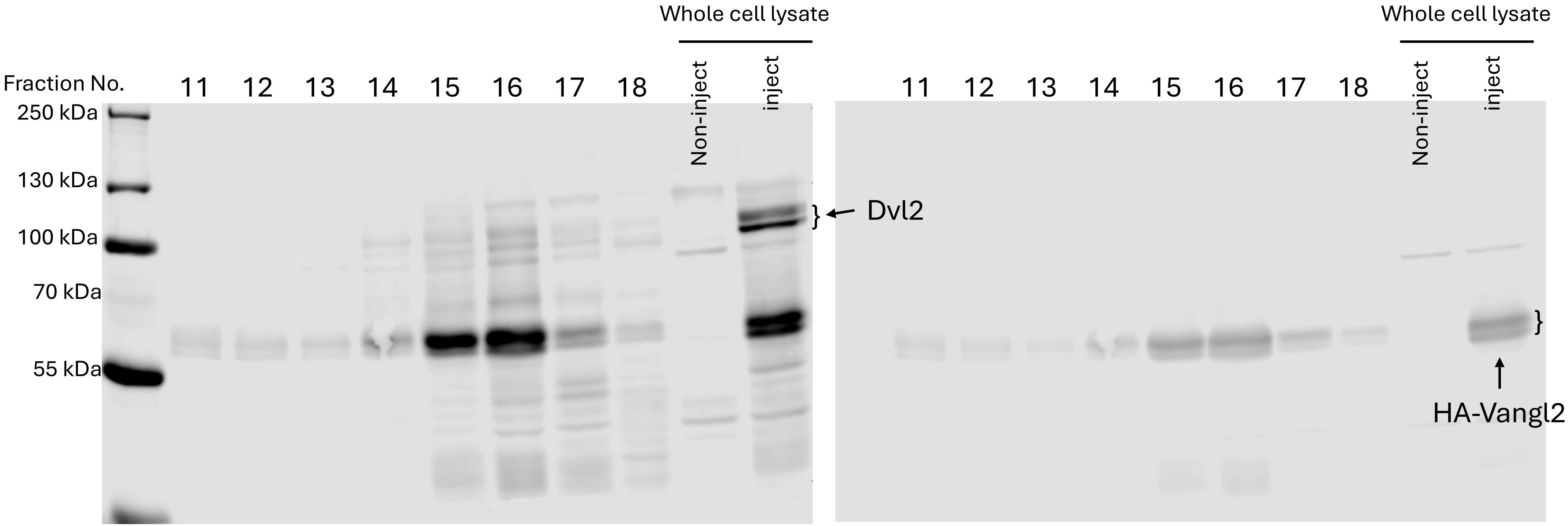

Whole cell lysate
Fraction No.
11
12
13
14
15
16
17
18
Non-inject
inject
250 kDa
130 kDa
100 kDa
70 kDa
55 kDa
Dvl2
Whole cell lysate
11
12
13
14
15
16
17
18
Non-inject
inject
HA-Vangl2
