# Supplementary material for: Prickle and Ror modulate Dishevelled-Vangl interaction to regulate non-canonical Wnt signaling during convergent extension in Xenopus
Source: eLife. 2026 Apr 30;12:RP91199. doi: 10.7554/eLife.91199 (PMC13132548; doi:10.7554/eLife.91199)
Supplement: MDAR checklist [file elife-91199-mdarchecklist1.docx]

**Materials Design Analysis Reporting (MDAR)**

**Checklist for Authors**

The [MDAR framework](https://osf.io/xfpn4/) establishes a minimum set of requirements in transparent reporting mainly applicable to studies in the life sciences.

*eLife* asks authors to **provide detailed information within their article** to facilitate the interpretation and replication of their work. Authors can also upload supporting materials to comply with relevant reporting guidelines for health-related research (see [EQUATOR Network](http://www.equator-network.org/%20)), life science research (see the [BioSharing Information Resource](http://biosharing.org/)), or animal research (see the [ARRIVE Guidelines](http://www.plosbiology.org/article/info:doi/10.1371/journal.pbio.1000412) and the [STRANGE Framework](https://doi.org/10.1038/d41586-020-01751-5); for details, see *eLife*’s [Journal Policies](https://reviewer.elifesciences.org/author-guide/journal-policies)). Where applicable, authors should refer to any relevant reporting standards materials in this form.

For all that apply, please note **where in the article** the information is provided. Please note that we also collect information about data availability and ethics in the submission form.

**Materials:**

| **Newly created materials** | **Indicate where provided: section/figure legend** | **N/A** |
| --- | --- | --- |
| The manuscript includes a dedicated "materials availability statement" providing transparent disclosure about availability of newly created materials including details on how materials can be accessed and describing any restrictions on access. |  | N/A |
|  |  |  |
| **Antibodies** | **Indicate where provided: section/figure legend** | **N/A** |
| Anti-FLAG M2 Magnetic Beads (Sigma Cat# 8823); 3 µg/ ml | Materials and Methods and relevant figure legend. |  |
| Anti-Myc Magnetic Beads (Pierce Cat.#88843); 3 µg/ ml | Materials and Methods and relevant figure legend. |  |
| Anti-GFP antibody (Santa Cruz Biotechnology (Cat# sc-9996) 1:500 western blot; | Materials and Methods and Figure Legends for Fig. 2g; Fig. 2 sup.1a,b,d; Fig2. Sup.4e; Fig. 3 sup.2a; Fig. 6d. |  |
| Anti-myc antibody (Santa Cruz Biotechnology (Cat# sc-373712) 1:1000 western blot; | Materials and Methods and Figure legend for Fig. 7 sup.2a,b |  |
| Anti-Dvl2 (CST Cat.# 3224) 1:1000 western blot; | Materials and Methods and Figure Legend for Fig. 7 sup.2a, c. |  |
| Anti-XRor2 (Developmental Studies Hybridoma Bank DA6D11) 0.5 µg/mL western blot; | Materials and Methods and Figure legend for Fig. 7 sup.2b,c |  |
| Anti-flag antibody (Sigma Cat# F1804) 1:1000 western blot; 1:200 immunofluorescence staining | Materials and Methods and Figure Legend Fig. 2g; Fig. 2 sup.1a,b,c,d; Fig. 2 sup.4e; Fig. 3 sup.2a; Fig. 6a,b,c,d,e. |  |
| Anti-α-Tubulin antibody (Cell Signaling Technology Cat. #3873; 1:1000 western blot | Materials and Methods and Figure Legend for Fig. 2 sup.1a,b,c,d; |  |
| **Anti-HA antibody (**Cell Signaling Technology Cat. # 3724; 1:1000 western blot | Materials and Methods and Figure Legend for Fig. 7 sup.2c |  |
|  |  |  |
| **DNA and RNA sequences** | **Indicate where provided: section/figure legend** | **N/A** |
| Xenopus Vangl2-morpholino (XVMO):  5’ CGTTGGCGGATTTGGGTCCCCCCGA 3’ | Materials and Methods |  |
| Ror2-morpholino (Xror2-MO)  5' GTCAGGCGAGGTAAGGGGCAACACT 3' | Materials and Methods |  |
| Pk-morpholino (XPkMO):  5' CTTCTGATCCATTTCCAAAGGCATG 3' | Materials and Methods |  |
|  |  |  |
| **Cell materials** | **Indicate where provided: section/figure legend** | **N/A** |
| 293T: ATCC cat. #CRL-3216 | Materials and Methods and Figure Legend for Fig. 2 sup.1d |  |
| Primary cultures: Provide species, strain, sex of origin, genetic modification status. |  | N/A |
|  |  |  |
| **Experimental animals** | **Indicate where provided: section/figure legend** | **N/A** |
| *Xenopus laevis*:  Embryos from *Xenopus laevis* were used in this study. No genetic modifications were applied. Because experiments were conducted at early embryonic stages, sex was not considered a relevant variable. Embryos were staged according to standard developmental tables (Nieuwkoop and Faber), and developmental stage rather than chronological age was used to define experimental timing. | Materials and Methods |  |
| Animal observed in or captured from the field: Provide species, sex, and age where possible. |  | N/A |
|  |  |  |
| **Plants and microbes** | **Indicate where provided: section/figure legend** | **N/A** |
| Plants: provide species and strain, ecotype and cultivar where relevant, unique accession number if available, and source (including location for collected wild specimens). |  | N/A |
| Microbes: provide species and strain, unique accession number if available, and source. |  | N/A |
|  |  |  |
| **Human research participants** | **Indicate where provided: section/figure legend) or state if these demographics were not collected** | **N/A** |
| If collected and within the bounds of privacy constraints report on age, sex, gender and ethnicity for all study participants. |  | N/A |

**Design:**

| **Study protocol** | **Indicate where provided: section/figure legend** | **N/A** |
| --- | --- | --- |
| If the study protocol has been pre-registered, provide DOI. For clinical trials, provide the trial registration number OR cite DOI. |  | N/A |
|  |  |  |
| **Laboratory protocol** | **Indicate where provided: section/figure legend** | **N/A** |
| Provide DOI OR other citation details if detailed step-by-step protocols are available. |  | N/A |
|  |  |  |
| **Experimental study design (statistics details) *** | | |
| **For in vivo studies: State whether and how the following have been done** | **Indicate where provided: section/figure legend. If it could have been done, but was not, write “not done”** | **N/A** |
| Sample size determination:  No formal statistical method was used to predetermine sample size. Sample sizes were selected based on standard practice in the field and prior experience with *Xenopus laevis* embryo assays, ensuring sufficient numbers to achieve reproducible, interpretable and/or statistically significant results across independent experiments. | MDAR checklist in Supplement. |  |
| Randomisation:  Embryos were randomly allocated to experimental groups following fertilization and dejellying to minimize selection bias. For explant imaging, fields of view were selected without bias, and regions with adequate signal-to-noise ratio were imaged according to consistent acquisition criteria. | MDAR checklist in Supplement. |  |
| Blinding:  Blinding was not performed during experimental manipulation or outcome assessment, as phenotypic differences were readily observable. However, objective criteria were used for phenotype classification to reduce potential bias. | MDAR checklist in Supplement. |  |
| Inclusion/exclusion criteria:  All normally developing embryos were included in the analysis. Embryos that were unfertilized, damaged during handling, or exhibited abnormal development unrelated to experimental manipulation were excluded. For confocal imaging, explants with poor integrity or inadequate signal quality were excluded from analysis. No additional exclusion criteria were applied. | MDAR checklist in Supplement. |  |
|  |  |  |
| **Sample definition and in-laboratory replication** | **Indicate where provided: section/figure legend** | **N/A** |
| State number of times the experiment was replicated in the laboratory:  All experiment were independently replicated using embryos derived from at least three separate fertilization batches. | Figures and figure legends. |  |
| Define whether data describe technical or biological replicates: For functional assays, Western blot analyses, and confocal imaging of explants, data are derived from biological replicates across three independent batches. Each batch represents an independent biological replicate. Within each confocal imaging experiment, multiple embryos or explants were analyzed per condition, which constitute technical replicates. | Figures and figure legends. |  |
|  |  |  |
| **Ethics** | **Indicate where provided: section/submission form** | **N/A** |
| Studies involving human participants: State details of authority granting ethics approval (IRB or equivalent committee(s), provide reference number for approval. |  | N/A |
| Studies involving experimental animals: State details of authority granting ethics approval (IRB or equivalent committee(s), provide reference number for approval:  Animal experiments were performed in agreement with the National Institutes of Health. *Xenopus laevis* adults were maintained according to the established protocols by the Institutional Animal Care and Use Committee at the University of Alabama at Birmingham, under Animal Project Number IACUC-22388. | Materials and Methods |  |
| Studies involving specimen and field samples: State if relevant permits obtained, provide details of authority approving study; if none were required, explain why. |  | N/A |
|  |  |  |
| **Dual Use Research of Concern (DURC)** | **Indicate where provided: section/submission form** | **N/A** |
| If study is subject to dual use research of concern regulations, state the authority granting approval and reference number for the regulatory approval. |  | N/A |

**Analysis:**

| **Attrition** | **Indicate where provided: section/figure legend** | **N/A** |
| --- | --- | --- |
| Describe whether exclusion criteria were pre-established. Report if sample or data points were omitted from analysis. If yes, report if this was due to attrition or intentional exclusion and provide justification:  No exclusion of data. | No exclusion of data. | N/A |
|  |  |  |
| **Statistics** | **Indicate where provided: section/figure legend** | **N/A** |
| Describe statistical tests used and justify choice of tests:  All statistical quantification analyses were performed using two-tailed Student’s *t*-tests to compare differences between two groups. And a significance threshold of *p* < 0.05 was used. | Statistics, sample size and replicates are described in each figure legend. |  |
|  |  |  |
| **Data availability** | **Indicate where provided: section/submission form** | **N/A** |
| For newly created and reused datasets, the manuscript includes a data availability statement that provides details for access (or notes restrictions on access). |  | N/A |
| When newly created datasets are publicly available, provide accession number in repository OR DOI and licensing details where available.  No new datasets; all relevant data and resources can be found within the article and its supplementary information. | Data availability statement | N/A |
| If reused data is publicly available provide accession number in repository OR DOI, OR URL, OR citation. |  | N/A |
|  |  |  |
| **Code availability** | **Indicate where provided: section/figure legend** | **N/A** |
| For any computer code/software/mathematical algorithms essential for replicating the main findings of the study, whether newly generated or re-used, the manuscript includes a data availability statement that provides details for access or notes restrictions. |  | N/A |
| Where newly generated code is publicly available, provide accession number in repository, OR DOI OR URL and licensing details where available. State any restrictions on code availability or accessibility. |  | N/A |
| If reused code is publicly available provide accession number in repository OR DOI OR URL, OR citation. |  | N/A |

**Reporting:**

The MDAR framework recommends adoption of discipline-specific guidelines, established and endorsed through community initiatives.

| **Adherence to community standards** | **Indicate where provided: section/figure legend** | **N/A** |
| --- | --- | --- |
| State if relevant guidelines (e.g., ICMJE, MIBBI, ARRIVE, STRANGE) have been followed, and whether a checklist (e.g., CONSORT, PRISMA, ARRIVE) is provided with the manuscript. | ICMJE guideline was followed, and MDAR checklist is provided in Supplement. |  |

* We provide the following guidance regarding transparent reporting and statistics; we also refer authors to [Ten common statistical mistakes to watch out for when writing or reviewing a manuscript](https://doi.org/10.7554/eLife.48175).

**Sample-size estimation**

- You should state whether an appropriate sample size was computed when the study was being designed
- You should state the statistical method of sample size computation and any required assumptions
- If no explicit power analysis was used, you should describe how you decided what sample (replicate) size (number) to use

**Replicates**

- You should report how often each experiment was performed
- You should include a definition of biological versus technical replication
- The data obtained should be provided and sufficient information should be provided to indicate the number of independent biological and/or technical replicates
- If you encountered any outliers, you should describe how these were handled
- Criteria for exclusion/inclusion of data should be clearly stated
- High-throughput sequence data should be uploaded before submission, with a private link for reviewers provided (these are available from both GEO and ArrayExpress)

**Statistical reporting**

- Statistical analysis methods should be described and justified
- Raw data should be presented in figures whenever informative to do so (typically when N per group is less than 10)
- For each experiment, you should identify the statistical tests used, exact values of N, definitions of center, methods of multiple test correction, and dispersion and precision measures (e.g., mean, median, SD, SEM, confidence intervals; and, for the major substantive results, a measure of effect size (e.g., Pearson's r, Cohen's d)
- Report exact p-values wherever possible alongside the summary statistics and 95% confidence intervals. These should be reported for all key questions and not only when the p-value is less than 0.05.

**Group allocation**

- Indicate how samples were allocated into experimental groups (in the case of clinical studies, please specify allocation to treatment method); if randomization was used, please also state if restricted randomization was applied
- Indicate if masking was used during group allocation, data collection and/or data analysis
